# Integrated lipidomics and fatty acid positional distribution for nutritional assessment of nine mammalian milks

**DOI:** 10.1038/s41538-026-00954-w

**Published:** 2026-07-03

**Authors:** Meiqing Chen, Songcui Lu, Lin Song, Jun Zhang, Yingjie Qi, Xiaowei Zhao, Nan Zheng, Yangdong Zhang, Jiaqi Wang

**Affiliations:** 1 Key Laboratory of Quality & Safety Control for Milk and Dairy Products of Ministry of Agriculture and Rural Affairs, Institute of Animal Science, Chinese Academy of Agricultural Sciences, Beijing, PR China; 2 Laboratory of Quality and Safety Risk Assessment for Dairy Products of Ministry of Agriculture and Rural Affairs, Institute of Animal Science, Chinese Academy of Agricultural Sciences, Beijing, PR China; 3 State Key Laboratory of Animal Nutrition and Feeding, Institute of Animal Science, Chinese Academy of Agricultural Sciences, Beijing, PR China

**Keywords:** Biochemistry, Chemical biology, Chemistry, Physiology

## Abstract

The nutritional value of milk fat depends on lipid and fatty acid composition. This study characterized the lipidomics and fatty acid positional distribution of milk from nine mammalian species: cow, buffalo, yak, goat, sheep, camel, mare, donkey, and human. Lipid molecular species were profiled by liquid chromatography–tandem mass spectrometry. Fatty acid positional distribution was determined using NH₂ and Si solid-phase extraction, selective sn-1/3 hydrolysis by *Candida antarctica lipase B*, and sn-2 monoacylglycerol quantification by gas chromatography–mass spectrometry. Ruminant milks showed high compositional similarity and differed from camel and monogastric milks. Compared to human milk, animal milks had higher triacylglycerols, phosphatidylcholines, phosphatidylethanolamines, and sphingomyelins, but lower free fatty acids and diacylglycerols. Short-chain fatty acids consistently occupied sn-1/3 positions, whereas medium-chain, long-chain, odd-chain, and branched-chain fatty acids favored sn-2 position. Monogastric milks exhibited sn-2 preference for palmitic acid and other saturated fatty acids and sn-1/3 preference for unsaturated fatty acids; ruminant and camel milks showed the reverse. Bray–Curtis analysis identified donkey milk as the closest lipidomic analog to human milk, followed by mare milk. These findings provide a systematic reference for evaluating mammalian milks and data for humanized infant formula lipid design.

## Introduction

Human milk is the gold standard for infant nutrition because it provides a balanced supply of energy, essential nutrients, and bioactive compounds tailored to early development^1^. When maternal supply is insufficient—owing to genetic, dietary, or other factors—infant formula supports growth. Despite efforts to align macro- and micronutrient profiles with nutrient requirements, formulas still differ from human milk in composition and molecular structure, so full replication remains difficult^2^. Milks from domesticated mammals (e.g., goat, sheep, camel, yak, buffalo, donkey, mare) offer alternative lipid sources that may reduce these gaps. Systematic, standardized comparisons across species can therefore guide the rational design of more humanized formulas.

Lipids contribute ~3.6% of human milk by mass yet provide 40–50% of infants’ dietary energy^3,4^. They also deliver fat-soluble vitamins and essential fatty acids (FA) and carry bioactive linked to immune protection, gut maturation, and neurodevelopment^3,5^. The milk lipidome is highly diverse, and hundreds of FA species have been reported; the theoretical variety of glycerolipid (GL), glycerophospholipid (GP), and sphingolipid (SP) molecules is correspondingly large^6^. Crucially, nutritional function depends on lipid molecular species and positional configuration, not merely totals—particularly FA forms (GL, GP and free)^7^. Studies have demonstrated the enhanced bioavailability of docosahexaenoic acid (DHA)when esterified in lysophosphatidylcholine (DHA-LysoPC) and in phosphatidylcholine at the sn-1 position (DHA-PC), compared to its triacylglycerol form (DHA-TG)^8,9^. Similarly, sn-2 palmitate improves palmitate and calcium bioavailability compared with sn-1/3 palmitate^10,11^. Medium-chain TGs are associated with weight management, with sn-1/3 medium-chain FA sometimes more effective than sn-2^12^. Thus, a thorough nutritional evaluation of milk demands an investigation that delves into the molecular specifics and stereospecific positioning of its lipid components.

Bovine milk exhibits a markedly different FA profile from human milk, characterized by higher saturated fatty acids (SFAs) and lower levels of omega-3 long-chain polyunsaturated fatty acids such as eicosapentaenoic acid (EPA) and DHA. This compositional discrepancy limits its suitability as a sole ingredient in infant formula^7,13^. In recent years, lipidomics studies have increasingly compared the lipid compositions of various mammalian milks—including ruminants (e.g., buffalo, yak, goat, camel) and non-ruminants (e.g., donkey, mare)—with human milk, aiming to identify alternative dairy sources with improved nutritional profiles^14–19^. These investigations have revealed notable interspecies differences in GL, GP, and fat globule architecture. Although detailed lipidomic data for individual species, such as bovine milk, are available, integrated multi-species analyses remain scarce^20,21^. Comparative interpretation across independent studies is hampered by analytical challenges, including the detection of low-abundance lipid species, wide dynamic range, and variations in instrumental platforms and data processing workflows^22^. Therefore, a harmonized analytical approach applied to a broad range of milk types is essential to enable reliable cross-species comparisons.

The regiospecific distribution of FAs in TG varies among mammalian species and influences the nutritional properties of milk lipids^23–25^. Available data on the sn-2 FA composition of human and bovine milk reveal distinct stereospecific patterns: in both human and bovine milk, short- and medium-chain FAs (SCFA and MCFA) are predominantly esterified at the sn-3 position. Long-chain FAs (LCFAs) are primarily located at the sn-2 position and are rarely found at sn-3. In contrast, monounsaturated FAs (MUFAs) and polyunsaturated FAs (PUFAs) are mainly distributed at the sn-1 and sn-3 positions. While MUFAs show similar esterification preference between the sn-1 and sn-3 positions, PUFAs exhibit a marked preference for the sn-3 over the sn-1 position^7,26^. Pancreatic lipase has been conventionally used for the partial hydrolysis of TGs in regiospecific analysis. However, its substrate selectivity introduces analytical bias, particularly for TGs rich in SCFAs, such as those in bovine milk^27,28^. To overcome this limitation, immobilized lipase from *Candida antarctica lipase B* (*CALB*) has been introduced as a more robust alternative and has been successfully applied to dairy lipids and other fats with high SCFA content^28–30^. Although detailed sn-positional profiles of bovine milk TGs based on *CALB* methodology are available^31^, comparable studies for other mammalian milk sources remain limited. Furthermore, the positional distributions of several minor FA classes—such as branched-chain FAs (BCFAs), odd-chain FAs (OCFAs), and SCFAs—which are increasingly recognized for their importance in human and ruminant health, have not been systematically investigated across different species.

Herein, we address these research gaps through an integrated lipidomics and fatty acidomics investigation of human milk (HM) and milk from eight mammalian species: Holstein cow (BOM), yak (YM), buffalo (BUM), goat (GM), sheep (SM), camel (CM), mare (MM), and donkey (DM). Specifically, we (i) characterize the milk lipidomes using LC–MS/MS; (ii) determine the sn-positional distribution of fatty acids via *CALB*-assisted regioselective hydrolysis followed by GC–MS analysis; and (iii) evaluate the compositional similarity of each milk type to human milk based on Bray–Curtis dissimilarity metrics. This coordinated analytical strategy provides harmonized cross-species datasets and extends sn-positional information—particularly for minor fatty acid classes such as BCFAs, OCFAs, and SCFAs. The resulting molecular and structural insights identify candidate lipid species and stereospecific motifs that may support the rational design of more human-like infant formulas.

## Results and discussion

### Differential lipid molecular species among mammalian milk

**Fig. 1:**
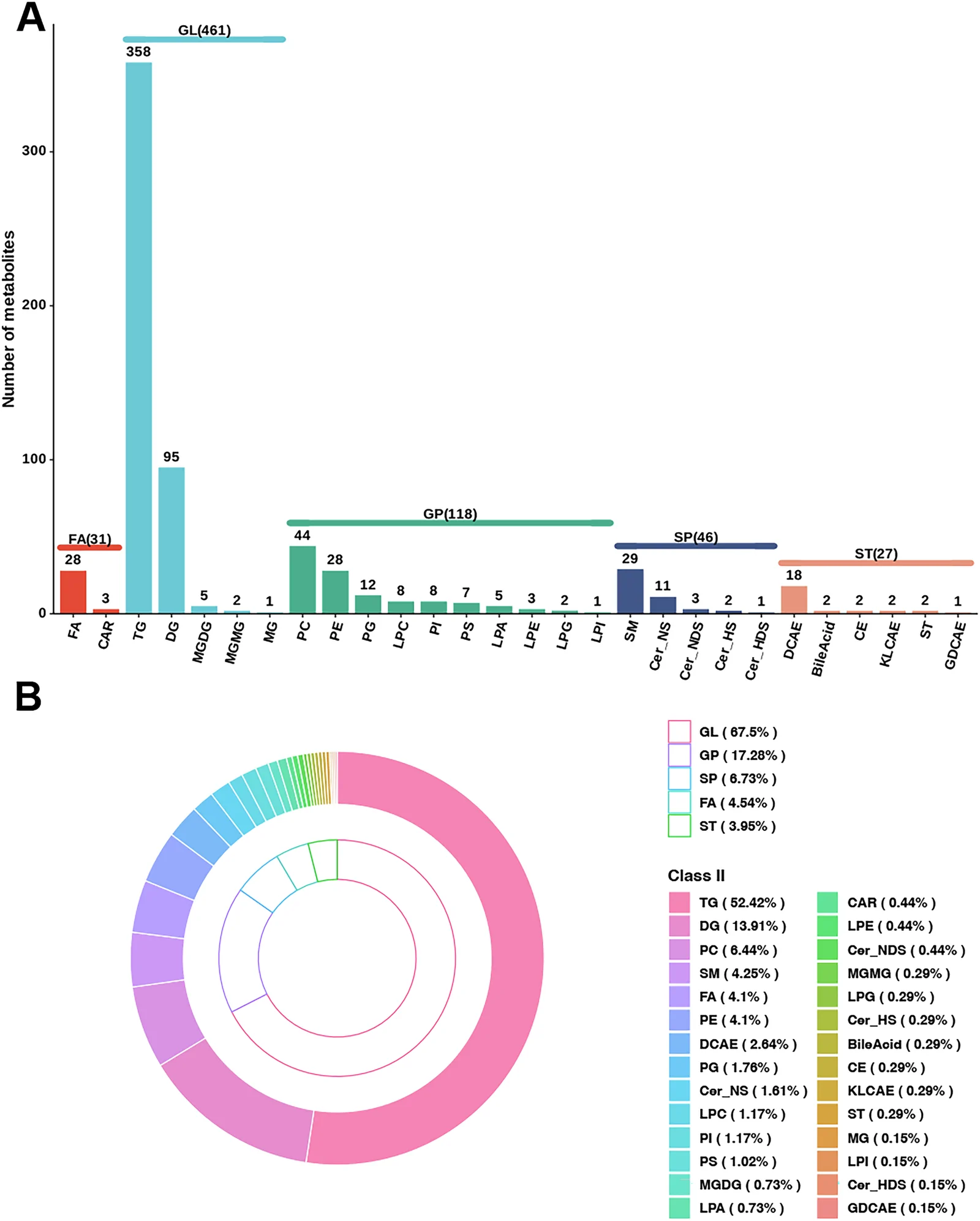
Lipid profiles of milks from nine mammalian species. **A** Numbers of lipid classes and subclasses. **B** Numbers percentage of lipid classes and subclasses.

Lipidomic analysis identified 683 lipid molecules across all milk samples, spanning five major categories (GL, GP, SP, ST, FFA) and 25 subclasses (Fig. 1A and Supplementary Data 1). GL was the predominant category, accounting for 67.5% (461 species) of the total lipidome, followed by GP (17.3%), SP (6.7%), FA (4.5%), and ST (4.0%) (Fig. 1B). At the subclass level, TG were the most abundant in all species, consistently followed by diacylglycerols (DG), phosphatidylcholines (PC), sphingomyelins (SM), and phosphatidylethanolamines (PE). Although minor variations in abundance ranking were observed, these five subclasses constituted the core lipid profile, consistent with established literature^16,32^.

**Fig. 2:**
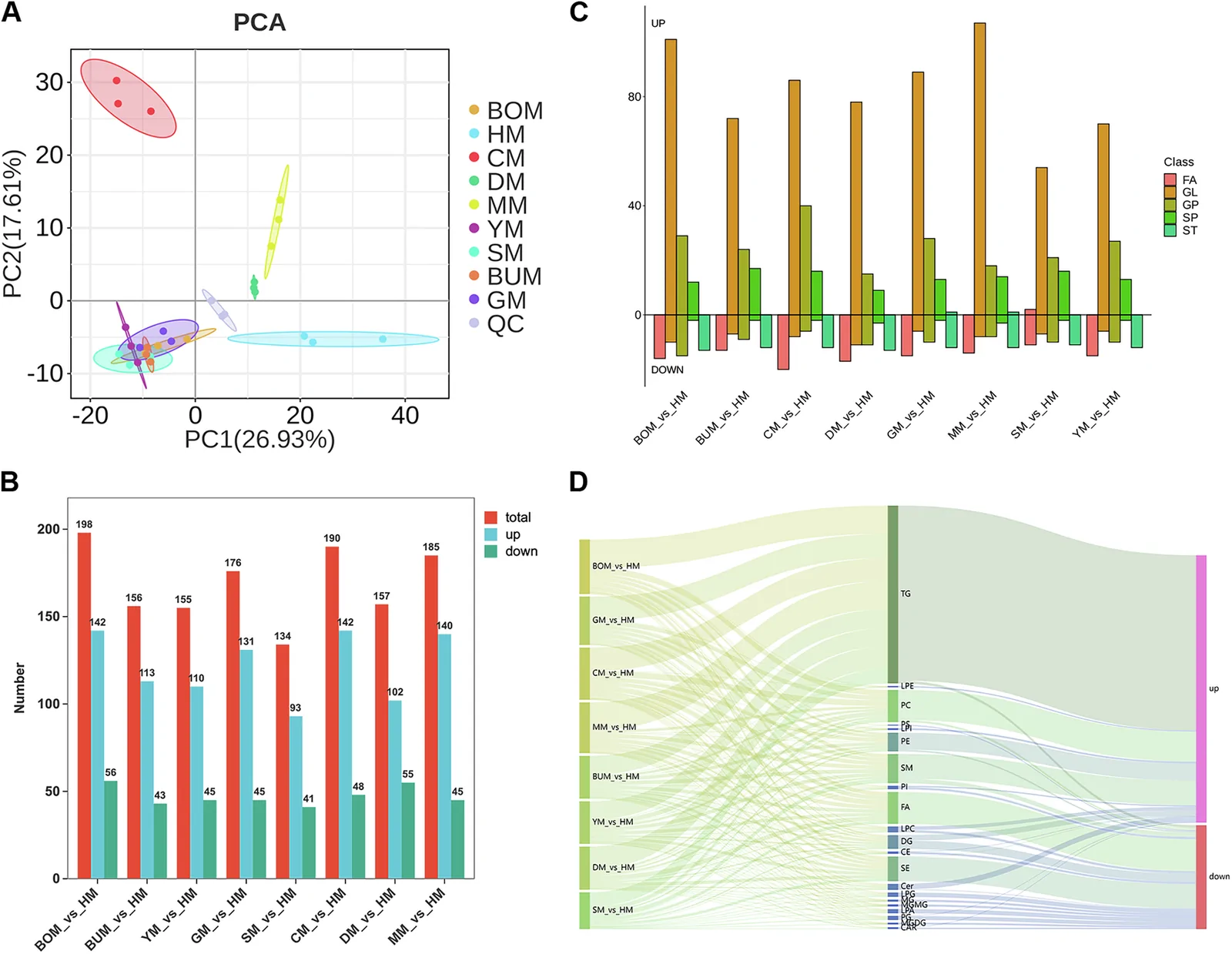
Pairwise comparative analysis of lipid composition between milk from eight animal species and human milk. **A** Scores plot of principal component analysis for nine animal milks. **B** Number of differential lipids molecules. **C** Distribution of lipid classes of differential lipids. **D** Distribution of lipid subclasses of differential lipids.

The score plot of PCA revealed species-specific clustering of milk lipid profiles (Fig. 2A). Ruminant milks (cow, buffalo, yak, goat, and sheep) showed partial overlap, indicating compositional similarity. In contrast, camel and monogastric milks (mare, donkey, human) formed distinct, well-separated clusters. The tight clustering of QC samples confirmed instrumental stability and data reliability. These lipidomic patterns align with phylogenetic relationships^33^. Specifically, the close genetic kinship of mare and donkey (family Equidae, order Perissodactyla) mirrors the high similarity of their milk lipidomes, distinct from Artiodactyla (cow, buffalo, yak, goat, sheep, camel). Within Artiodactyla, the distinct lipid profile of camel milk (Family Camelidae) separates it from bovid milks (Family Bovidae), while within Bovidae, subfamily groupings (Bovinae vs. Caprinae) correspond to the observed compositional overlap. These results suggest that genetic factors are a primary determinant of interspecies variation in milk lipid composition, consistent with previous reports^16^.

To identify specific lipid molecules responsible for these differences, differential lipids (DLs) were defined using FC > 2 or <0.5, *P* < 0.05, and VIP > 1 from the OPLS-DA model, with human milk as the control. As shown in Fig. 2B, the number of DLs identified in comparisons with cow, buffalo, yak, goat, sheep, camel, mare, and donkey milk were 198, 156, 155, 176, 134, 190, 185, and 157, respectively. Notably, most differential species in animal milks exhibited higher relative abundance compared to human milk. At the category level, upregulated lipids in animal milks primarily belonged to GL, GP, and SP, while downregulated lipids were predominantly FA, with additional contributions from GL, GP, and ST (Fig. 2C). A Sankey diagram illustrated that TG, DG, PC, PE, SM, and FFA constituted the major differential lipid subclasses (Fig. 2D). Most of these DLs were upregulated in non-human milks relative to human milk. This trend corroborates Wu et al.^32^, who noted higher relative abundances of specific lipid species in ruminant milks. The unique rumen microbial ecosystem, which ferments high-fiber diets and biohydrogenates fatty acids such as linoleic acid to CLA^34,35^, likely contributes to these distinct lipid profiles compared to monogastric human milk.

**Fig. 3:**
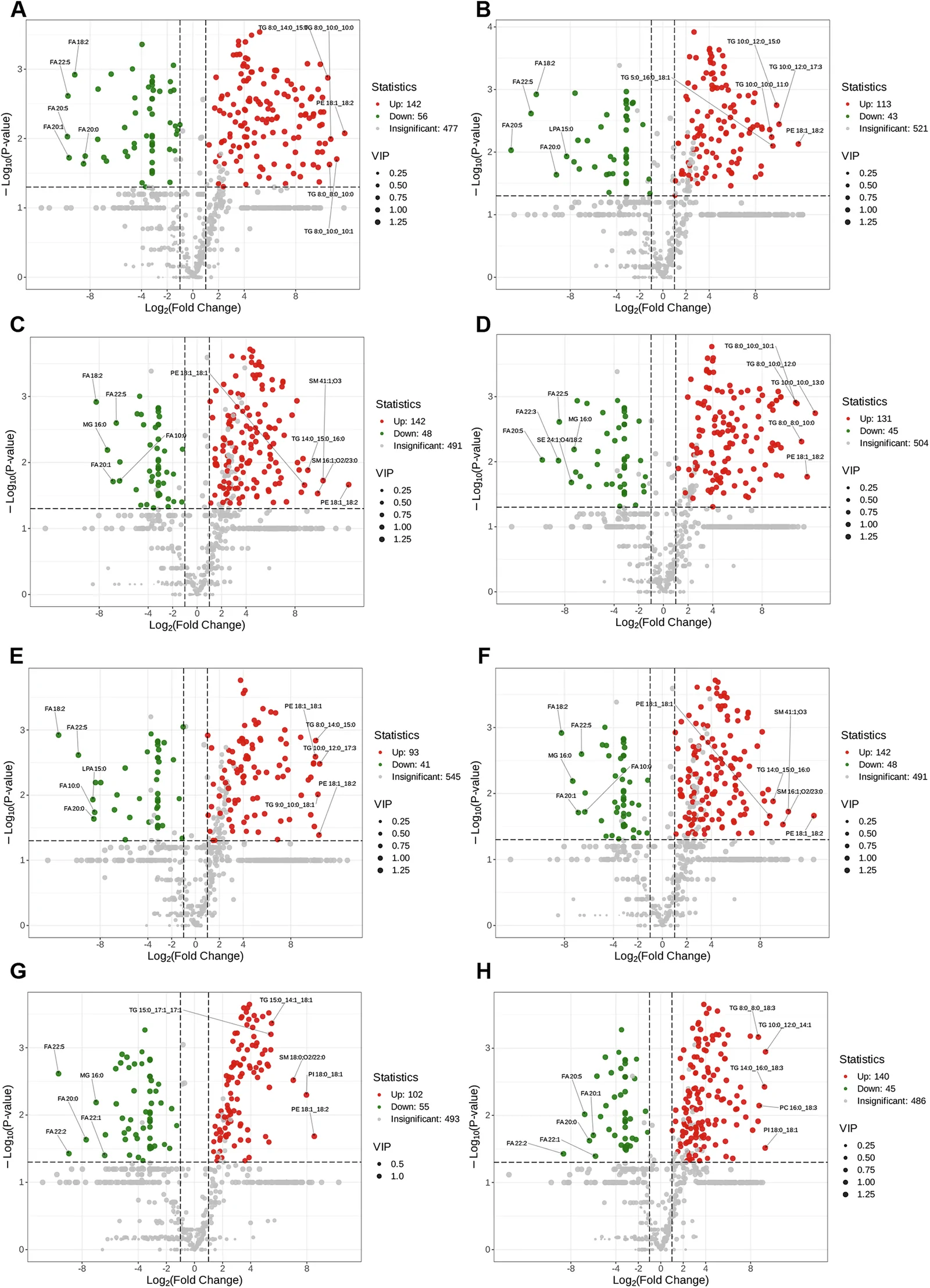
Volcano plots of differential lipids in pairwise comparisons between eight mammalian milks and human milk. **A** Holstein cow vs. human milk. **B** Buffalo vs. human milk. **C** Yak vs. human milk. **D** Goat vs. human milk. **E** Sheep vs. human milk. **F** Camel vs. human milk. **G** Donkey vs. human milk. **H** Mare vs. human milk.

These patterns were further validated by OPLS-DA, which confirmed significant lipid disparities among the groups (Supplementary Fig. 1A–H). Volcano plots further detailed the most significantly altered lipid species in each comparison (Fig. 3 and Supplementary Data 2). In Holstein cow milk versus human milk, elevated species included PE 36:3 and TG 28:1, while FA 20:0 and FA 18:2 were reduced (Fig. 3A). Buffalo milk showed elevated PE 36:3 and TG 39:3, with reduced LPA 15:0 and FA 20:0 (Fig. 3B). Yak milk uniquely featured elevated SM 33:1 alongside TG and PE (Fig. 3C). Goat milk exhibited elevated TG 33:0 and reduced MG 16:0 and SE 42:1 (Fig. 3D). Sheep milk showed elevated TG 39:3 and reduced LPA 15:0 (Fig. 3E). Camel milk displayed elevated SM 41:1 and PE 36:3 (Fig. 3F). Both donkey and mare milk were characterized by elevated PI 36:1 and unique TG profiles (e.g., TG 47:2 in donkey milk; TG 36:1 in mare milk) alongside reduced FA 22:1 (Fig. 3G, H). These findings are broadly consistent with Zhao et al.^16^, who identified TG and DG as the primary differential lipid subclasses between human and ruminant milks.

**Fig. 4:**
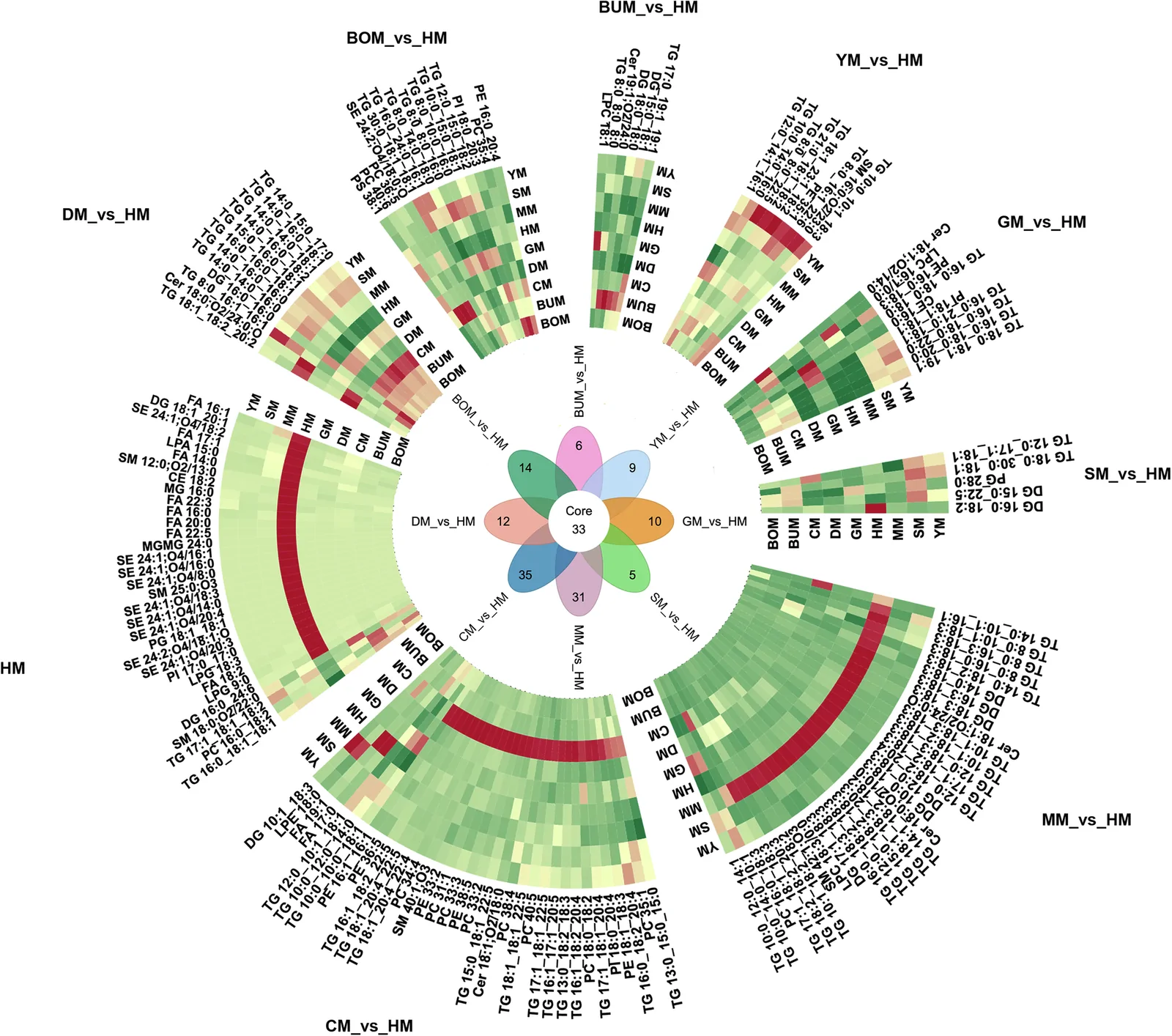
Venn diagram and cluster heatmap of differential lipids between eight mammalian milks and human milk.

Venn diagram analysis revealed unique and shared differential lipids across the eight comparisons (Fig. 4). Unique species with elevated abundance were identified for specific milks, including PI 18:0_20:3 in cow milk; TG 8:0_8:0_8:0 in buffalo milk; TG 10:0_14:0_16:0 in yak milk; CE 16:0 in goat milk; TG 12:0_17:1_18:1 in sheep milk; and Cer 18:0;O2/24:0;O in donkey milk. Notably, camel milk exhibited 35 unique differential lipids, predominantly PC and PE species containing C18 and C20 fatty acyl chains. Conversely, 33 common differential lipids were shared across all eight comparisons, primarily belonging to FA and SE classes. This indicates that while each mammalian milk possesses a species-specific lipid signature, a universal divergence from human milk exists in the FFA and sterol ester profiles.

The observed upregulation of TG and PE species in mammalian milks likely reflects an evolutionary adaptation to meet the high energy and membrane synthesis demands of rapidly growing neonates^36^. Conversely, the downregulation of FFA species may indicate distinct mechanisms of lipid mobilization and absorption compared to humans. The enrichment of SM and PI in specific milks (camel, donkey, mare) may confer specialized benefits for neurodevelopment and cell signaling^37^. In summary, the species-specific variation in milk lipid composition results from a combination of mammary gland evolution, neonatal nutritional requirements, and dietary physiology. The similarity among ruminant milks reflects their shared digestive anatomy and ruminal fermentation, whereas the distinct signatures of non-ruminant milks underscore their unique ecological and lactational adaptations^38,39^.

### Regiospecific distribution of fatty acid among mammalian milk

A comprehensive analysis of the regiospecific distribution of FAs revealed fundamental structural differences in TGs among the studied mammalian milks (Tables 1–3). At the overall TG level, human (37.62 mol%), donkey (40.05 mol%), and mare milk (50.22 mol%) formed a distinct cluster characterized by a moderate content of even-chain saturated fatty acids (ECSFA), which was substantially lower than that of Holstein cow (69.87 mol%) and goat milk (69.63 mol%). Camel milk (60.97 mol%) occupied an intermediate position. This trend was consistent at the positional level: at the sn-2 position, human (50.76 mol%) and donkey milk (52.32 mol%) exhibited similar ECSFA levels, which contrasted sharply with the elevated values in Holstein cow (69.31 mol%) and goat milk (72.05 mol%). Consequently, human, donkey, and mare milk displayed a clear preference for esterifying ECSFA at the sn-2 position, whereas in buffalo, yak, sheep, and camel milk, ECSFA were predominantly located at the sn-1/3 positions.

**Table 1:** Variation in the total fatty acids across eight mammalian species and human milk fat triacylglycerols

Fatty acid	TG (mol%)									
	BOM	BUM	YM	GM	SM	CM	DM	MM	HM	*P*-value
SFA										
ECSFA										
C4:0	8.79	17.93	15.34	6.08	11.25	0.12	0.55	0.34	0.07	<0.01
	(0.89)	(1.06)	(2.21)	(0.23)	(1.03)	(0.01)	(0.09)	(0.06)	(0.002)	
C6:0	4.38	5.08	7.61	4.23	6.67	0.51	0.76	0.48	0.08	<0.01
	(0.43)	(0.42)	(1.75)	(0.16)	(0.49)	(0.02)	(0.16)	(0.001)	(0.01)	
C8:0	1.70	0.76	1.56	2.75	2.47	0.19	4.62	3.72	0.22	<0.01
	(0.02)	(0.08)	(0.26)	(0.19)	(0.45)	(0.02)	(0.15)	(0.45)	(0.01)	
C10:0	3.11	1.30	2.22	10.26	6.32	0.21	6.74	6.78	1.48	<0.01
	(0.04)	(0.13)	(0.40)	(0.74)	(0.87)	(0.01)	(0.30)	(0.54)	(0.07)	
C12:0	3.18	1.53	1.64	5.13	3.40	1.19	4.57	6.77	3.92	<0.01
	(0.05)	(0.21)	(0.35)	(0.45)	(0.27)	(0.05)	(0.25)	(0.66)	(0.37)	
C14:0	9.85	6.43	6.63	9.52	9.57	15.25	3.21	7.32	3.70	<0.01
	(0.45)	(0.58)	(0.63)	(0.78)	(0.45)	(0.86)	(0.12)	(0.42)	(0.03)	
C16:0	31.08	26.35	24.03	27.02	23.36	26.45	18.86	24.23	22.04	<0.01
	(1.42)	(1.72)	(0.76)	(0.66)	(0.58)	(0.98)	(0.19)	(0.14)	(1.01)	
C18:0	7.71	11.20	12.62	4.27	9.18	16.78	0.71	0.55	6.00	<0.01
	(0.18)	(0.79)	(0.72)	(0.06)	(0.92)	(1.02)	(0.03)	(0.06)	(0.82)	
C20:0	0.04	0.16	0.24	0.15	0.20	0.18	0.02	0.03	0.07	<0.01
	(0.002)	(0.02)	(0.06)	(0.01)	(0.05)	(0.02)	(0.001)	(0.003)	(0.003)	
C22:0	0.01	0.06	0.05	0.14	0.04	0.07	0.01	NA	0.03	<0.01
	(0.001)	(0.01)	(0.01)	(0.01)	(0.001)	(0.01)	(0.002)	NA	(0.001)	
C24:0	0.01	NA	0.03	0.07	0.02	0.02	NA	NA	0.01	<0.01
	(0.001)	NA	(0.005)	(0.005)	(0.002)	(0.0003)	NA	NA	(0.0004)	
OCSFA										
C5:0	0.07	0.12	0.04	0.04	0.06	NA	NA	NA	NA	<0.01
	(0.01)	(0.001)	(0.004)	(0.003)	(0.02)	NA	NA	NA	NA	
C7:0	0.04	0.01	0.01	0.03	0.03	NA	NA	0.01	NA	<0.01
	(0.002)	(0.002)	(0.002)	(0.001)	(0.005)	NA	NA	(0.001)	NA	
C9:0	0.04	0.02	0.01	0.06	0.05	0.01	0.04	0.02	0.01	<0.01
	(0.002)	(0.001)	(0.0004)	(0.003)	(0.01)	(0.001)	(0.002)	(0.003)	(0.001)	
C11:0	0.07	0.02	0.01	0.06	0.06	0.03	0.03	0.01	0.01	<0.01
	(0.002)	(0.002)	(0.002)	(0.01)	(0.01)	(0.001)	(0.002)	(0.003)	(0.001)	
C13:0	0.09	0.07	0.05	0.09	0.09	0.09	0.02	0.02	0.02	<0.01
	(0.004)	(0.01)	(0.01)	(0.003)	(0.01)	(0.01)	(0.002)	(0.001)	(0.002)	
C15:0	0.77	0.76	1.07	0.52	0.79	1.16	0.15	0.15	0.15	<0.01
	(0.01)	(0.03)	(0.10)	(0.06)	(0.03)	(0.05)	(0.01)	(0.005)	(0.03)	
C17:0	0.39	0.32	0.69	0.33	0.50	0.50	0.11	0.08	0.17	<0.01
	(0.01)	(0.01)	(0.09)	(0.02)	(0.03)	(0.03)	(0.001)	(0.002)	(0.03)	
C21:0	0.004	0.02	0.03	0.16	0.02	0.11	NA	NA	0.003	<0.01
	(0.0001)	(0.0018)	(0.01)	(0.01)	(0.001)	(0.004)	NA	NA	(0.0004)	
C23:0	0.01	0.04	0.03	0.08	0.02	0.04	NA	NA	0.002	<0.01
	(0.001)	(0.003)	(0.004)	(0.004)	(0.001)	(0.01)	NA	NA	(0.0002)	
BCSFA										
iso C13:0	0.01	0.02	0.04	0.03	0.03	0.05	0.004	0.01	0.002	<0.01
	(0.001)	(0.002)	(0.001)	(0.001)	(0.001)	(0.004)	(0.0004)	(0.002)	(0.0003)	
anteiso C13:0	0.003	0.01	0.01	0.01	0.01	0.01	NA	NA	NA	<0.01
	(0.0001)	(0.0012)	(0.0013)	(0.0002)	(0.0006)	(0.0014)	NA	NA	NA	
iso C14:0	0.05	0.07	0.16	0.07	0.11	0.14	0.01	0.04	0.01	<0.01
	(0.001)	(0.005)	(0.01)	(0.002)	(0.01)	(0.01)	(0.0001)	(0.01)	(0.003)	
iso C15:0	0.10	0.15	0.24	0.08	0.19	0.29	0.01	0.01	0.02	<0.01
	(0.003)	(0.02)	(0.01)	(0.003)	(0.01)	(0.02)	(0.0005)	(0.001)	(0.01)	
anteiso C15:0	0.22	0.26	0.42	0.14	0.34	0.64	0.01	NA	0.03	<0.01
	(0.005)	(0.01)	(0.04)	(0.02)	(0.01)	(0.04)	(0.003)	NA	(0.01)	
iso C16:0	0.12	0.14	0.25	0.18	0.26	0.30	0.04	0.09	0.02	<0.01
	(0.003)	(0.02)	(0.02)	(0.01)	(0.02)	(0.01)	(0.001)	(0.01)	(0.01)	
iso C17:0	0.14	0.12	0.20	0.12	0.15	0.26	NA	0.01	0.02	<0.01
	(0.003)	(0.01)	(0.03)	(0.01)	(0.02)	(0.02)	NA	(0.0003)	(0.01)	
anteiso C17:0	0.23	0.18	0.36	0.19	0.33	0.55	0.04	0.12	0.06	<0.01
	(0.003)	(0.01)	(0.05)	(0.02)	(0.01)	(0.03)	(0.001)	(0.01)	(0.02)	
iso C18:0	0.02	0.02	0.03	0.05	0.04	0.07	0.01	0.02	NA	<0.01
	(0.001)	(0.003)	(0.004)	(0.001)	(0.01)	(0.002)	(0.001)	(0.001)	NA	
UFA										
MUFA										
C10:1 c9	0.26	0.01	0.14	0.24	0.19	0.03	0.95	1.20	0.005	<0.01
	(0.01)	(0.001)	(0.03)	(0.01)	(0.01)	(0.0001)	(0.05)	(0.34)	(0.0002)	
C14:1 c9	0.86	0.29	0.17	0.23	0.14	0.59	0.10	0.32	0.07	<0.01
	(0.02)	(0.01)	(0.03)	(0.02)	(0.03)	(0.10)	(0.01)	(0.07)	(0.01)	
C16:1 t9	NA	0.13	0.09	0.11	0.05	0.16	NA	NA	NA	<0.01
	NA	(0.01)	(0.01)	(0.02)	(0.01)	(0.03)	NA	NA	NA	
C16:1 c7	0.14	0.19	0.21	0.24	0.22	0.50	0.59	0.20	0.22	<0.01
	(0.01)	(0.01)	(0.03)	(0.003)	(0.03)	(0.05)	(0.02)	(0.02)	(0.02)	
C16:1 c9	1.82	0.86	0.92	0.72	0.74	4.83	2.23	5.01	1.22	<0.01
	(0.08)	(0.01)	(0.24)	(0.04)	(0.06)	(0.57)	(0.04)	(0.37)	(0.05)	
C17:1 c10	NA	0.11	NA	0.19	NA	0.31	NA	NA	NA	<0.01
	NA	(0.01)	NA	(0.01)	NA	(0.02)	NA	NA	NA	
C18:1 t6	0.17	0.30	0.12	0.22	0.16	0.56	0.01	NA	0.03	<0.01
	(0.02)	(0.01)	(0.003)	(0.01)	(0.04)	(0.03)	(0.001)	NA	(0.01)	
C18:1 t9	NA	0.15	0.11	0.22	0.13	0.23	0.03	NA	0.03	<0.01
	NA	(0.01)	(0.01)	(0.02)	(0.01)	(0.02)	(0.01)	NA	(0.01)	
C18:1 t11	0.43	3.01	1.83	0.44	0.56	1.31	0.01	NA	0.08	<0.01
	(0.003)	(0.28)	(0.31)	(0.09)	(0.08)	(0.47)	(0.001)	NA	(0.05)	
C18:1 c6	0.20	0.22	0.06	0.15	0.14	0.38	0.01	NA	0.03	<0.01
	(0.01)	(0.02)	(0.02)	(0.01)	(0.02)	(0.04)	(0.001)	NA	(0.01)	
C18:1 c9	19.50	17.60	17.51	18.57	19.43	19.66	27.72	18.63	28.64	<0.01
	(0.51)	(0.58)	(2.71)	(1.01)	(0.38)	(1.33)	(0.42)	(0.44)	(0.65)	
C18:1 c11	0.93	0.70	0.40	0.89	0.49	1.09	0.66	0.64	1.27	<0.01
	(0.07)	(0.03)	(0.13)	(0.02)	(0.26)	(0.17)	(0.06)	(0.05)	(0.07)	
C18:1 c12	0.22	0.14	0.06	0.10	0.16	0.21	0.04	NA	0.11	<0.01
	(0.01)	(0.02)	(0.03)	(0.004)	(0.02)	(0.01)	(0.01)	NA	(0.01)	
C20:1 t11	NA	NA	NA	NA	0.01	NA	NA	NA	NA	<0.01
	NA	NA	NA	NA	(0.002)	NA	NA	NA	NA	
C20:1 c8	0.04	0.07	0.10	NA	NA	0.06	NA	NA	NA	<0.01
	(0.003)	(0.005)	(0.02)	NA	NA	(0.002)	NA	NA	NA	
C20:1 c11	NA	NA	NA	0.11	0.03	0.11	0.17	0.53	0.18	<0.01
	NA	NA	NA	(0.01)	(0.001)	(0.003)	(0.01)	(0.07)	(0.005)	
C22:1 t13	NA	NA	NA	NA	NA	NA	NA	NA	NA	/
	NA	NA	NA	NA	NA	NA	NA	NA	NA	
C22:1 c13	NA	NA	NA	0.18	NA	0.08	NA	NA	NA	<0.01
	NA	NA	NA	(0.02)	NA	(0.001)	NA	NA	NA	
C24:1 c15	NA	NA	0.01	NA	0.01	NA	0.06	NA	0.01	<0.01
	NA	NA	(0.001)	NA	(0.001)	NA	(0.05)	NA	(0.001)	
PUFA										
C18:2 t9t12	NA	NA	NA	0.20	NA	0.12	NA	NA	NA	<0.01
	NA	NA	NA	(0.01)	NA	(0.004)	NA	NA	NA	
C18:2 c9t12	0.03	0.10	0.05	0.06	0.04	0.07	0.02	0.03	0.04	<0.01
	(0.002)	(0.03)	(0.03)	(0.01)	(0.01)	(0.01)	(0.002)	(0.001)	(0.002)	
C18:2 t9c12	NA	0.13	NA	NA	0.02	0.09	NA	NA	0.03	<0.01
	NA	(0.02)	NA	NA	(0.001)	(0.01)	NA	NA	(0.004)	
C18:2 c9c12	2.09	1.05	1.06	2.24	1.24	1.49	25.67	4.76	26.42	<0.01
	(0.11)	(0.15)	(0.21)	(0.87)	(0.10)	(0.30)	(0.61)	(0.21)	(2.01)	
C18:3 c6c9c12	0.02	0.03	0.003	0.19	0.03	0.13	0.01	0.06	0.06	<0.01
	(0.001)	(0.01)	(0.0002)	(0.01)	(0.02)	(0.004)	(0.001)	(0.02)	(0.001)	
C18:3 c9c12c15	0.19	0.16	0.58	0.30	0.15	0.60	0.83	16.75	1.79	<0.01
	(0.02)	(0.04)	(0.15)	(0.05)	(0.07)	(0.06)	(0.02)	(2.38)	(0.16)	
C18:2 c9t11	0.24	0.93	0.63	0.30	0.26	0.35	0.03	0.03	0.05	<0.01
	(0.02)	(0.09)	(0.16)	(0.05)	(0.02)	(0.06)	(0.002)	(0.001)	(0.01)	
C18:2 c9c11	NA	0.25	NA	NA	NA	0.24	NA	NA	NA	<0.01
	NA	(0.02)	NA	NA	NA	(0.06)	NA	NA	NA	
C20:2 c11c14	0.01	NA	0.01	NA	0.004	0.05	0.17	NA	0.12	<0.01
	(0.001)	NA	(0.001)	NA	(0.001)	(0.002)	(0.02)	NA	(0.003)	
C20:3 c8c11c14	0.51	0.35	0.09	1.42	0.11	1.06	0.10	0.86	1.01	<0.01
	(0.01)	(0.06)	(0.02)	(0.08)	(0.01)	(0.03)	(0.001)	(0.07)	(0.04)	
C20:3 c11c14c17	NA	0.03	0.02	NA	NA	0.04	0.04	0.07	0.02	<0.01
	NA	(0.003)	(0.01)	NA	NA	(0.001)	(0.01)	(0.01)	(0.003)	
C20:4 c5c8c11c14	0.06	0.04	0.04	0.26	0.07	0.20	0.02	0.04	0.25	<0.01
	(0.005)	(0.005)	(0.01)	(0.01)	(0.004)	(0.01)	(0.001)	(0.003)	(0.02)	
C22:2 c13c16	NA	NA	NA	NA	NA	NA	NA	NA	0.01	<0.01
	NA	NA	NA	NA	NA	NA	NA	NA	(0.001)	
C20:5 c5c8c11c14c17	0.01	NA	0.02	0.14	0.01	0.08	NA	NA	0.02	<0.01
	(0.0003)	NA	(0.01)	(0.01)	(0.0002)	(0.02)	NA	NA	(0.01)	
C22:3 c13c16c19	NA	NA	NA	NA	NA	NA	NA	0.03	NA	<0.01
	NA	NA	NA	NA	NA	NA	NA	(0.002)	NA	
C22:4 c7c10c13c16	0.01	NA	0.004	0.14	0.01	0.10	NA	NA	0.03	<0.01
	(0.0004)	NA	(0.001)	(0.01)	(0.001)	(0.004)	NA	NA	(0.001)	
C22:5 c4c7c10c13c16	0.002	NA	0.001	0.13	0.01	NA	NA	NA	0.03	<0.01
	(0.0001)	NA	(0.0001)	(0.01)	(0.0003)	NA	NA	NA	(0.01)	
C22:5 c7c10c13c16c19	0.01	0.01	0.03	0.07	0.01	0.06	NA	NA	0.02	<0.01
	(0.0001)	(0.001)	(0.01)	(0.003)	(0.001)	(0.01)	NA	NA	(0.002)	
C22:6 c4c7c10c13c16c19	NA	NA	0.004	0.08	0.003	NA	NA	NA	0.07	<0.01
	NA	NA	(0.001)	(0.004)	(0.001)	NA	NA	NA	(0.01)	
FA group										
SFA	72.25	73.14	75.73	71.86	75.59	65.21	40.53	50.82	38.14	<0.01
	(0.81)	(0.73)	(3.79)	(1.91)	(0.78)	(1.49)	(0.98)	(2.11)	(1.46)	
ECSFA	69.87	70.80	71.98	69.63	72.49	60.97	40.05	50.22	37.62	<0.01
	(0.85)	(0.70)	(4.08)	(1.82)	(0.84)	(1.43)	(0.97)	(2.10)	(1.36)	
OCSFA	1.50	1.38	1.95	1.36	1.61	1.93	0.35	0.29	0.36	<0.01
	(0.04)	(0.04)	(0.18)	(0.08)	(0.05)	(0.03)	(0.01)	(0.01)	(0.06)	
BCSFA	0.88	0.96	1.81	0.87	1.5	2.31	0.13	0.31	0.16	<0.01
	(0.01)	(0.06)	(0.13)	(0.05)	(0.06)	(0.10)	(0.001)	(0.001)	(0.06)	
UFA	27.75	26.86	24.27	28.14	24.41	34.79	59.47	49.18	61.86	<0.01
	(0.81)	(0.73)	(3.79)	(1.91)	(0.78)	(1.49)	(0.98)	(2.11)	(1.46)	
n-1 UFA	0.26	0.01	0.14	0.24	0.19	0.03	0.95	1.20	0.005	<0.01
	(0.01)	(0.001)	(0.03)	(0.01)	(0.01)	(0.003)	(0.05)	(0.34)	(0.0002)	
n-3 UFA	0.21	0.20	0.65	0.59	0.16	0.78	0.87	16.85	1.93	<0.01
	(0.02)	(0.04)	(0.17)	(0.07)	(0.07)	(0.08)	(0.03)	(2.38)	(0.15)	
n-5 UFA	0.86	0.29	0.17	0.23	0.14	0.59	0.10	0.32	0.07	<0.01
	(0.02)	(0.01)	(0.03)	(0.02)	(0.03)	(0.10)	(0.01)	(0.07)	(0.01)	
n-6 UFA	2.95	1.84	1.31	4.74	1.7	3.51	26.03	5.75	28.10	<0.01
	(0.13)	(0.18)	(0.29)	(1.00)	(0.11)	(0.32)	(0.60)	(0.13)	(1.98)	
n-7 UFA	3.42	5.98	3.87	2.65	2.10	8.30	2.93	5.69	2.62	<0.01
	(0.15)	(0.32)	(0.77)	(0.13)	(0.30)	(0.19)	(0.09)	(0.37)	(0.07)	
n-9 UFA	19.64	17.95	17.84	19.31	19.82	20.57	28.57	19.37	29.08	<0.01
	(0.52)	(0.58)	(2.75)	(1.04)	(0.40)	(1.36)	(0.38)	(0.51)	(0.67)	
n-12 UFA	0.41	0.59	0.28	0.38	0.30	1.01	0.01	NA	0.06	<0.01
	(0.03)	(0.03)	(0.03)	(0.02)	(0.06)	(0.04)	(0.001)	NA	(0.02)	
MUFA	24.58	23.79	21.73	22.61	22.44	30.11	32.57	26.55	31.89	<0.01
	(0.66)	(0.72)	(3.33)	(1.09)	(0.70)	(1.72)	(0.43)	(0.60)	(0.74)	
PUFA	3.17	3.08	2.54	5.53	1.97	4.68	26.9	22.63	29.96	<0.01
	(0.17)	(0.22)	(0.51)	(1.11)	(0.14)	(0.35)	(0.56)	(2.26)	(2.13)	
SCFA	13.25	23.13	23.08	10.34	18.03	0.63	1.31	0.82	0.15	<0.01
	(1.34)	(1.44)	(3.97)	(0.39)	(1.53)	(0.01)	(0.25)	(0.06)	(0.01)	
MCFA	18.42	10.24	12.48	28.27	22.33	17.20	20.21	25.89	9.37	<0.01
	(0.56)	(0.65)	(1.61)	(2.15)	(1.90)	(0.96)	(0.87)	(2.39)	(0.41)	
LCFA	68.34	66.64	64.43	61.39	59.64	82.17	78.48	73.29	90.48	<0.01
	(0.78)	(1.54)	(5.54)	(2.38)	(0.70)	(0.97)	(1.12)	(2.44)	(0.41)	

Values without parentheses are means, and values in parentheses are standard errors. Fatty acid classification: SCFA (short-chain fatty acids, C1–C6); MCFA (medium-chain fatty acids, C7–C14); LCFA (long-chain fatty acids, C15–C24). NA indicates values below the limit of quantification.

*BOM* Holstein cow milk, *BUM* buffalo milk, *YM* yak milk, *GM* goat milk, *SM* sheep milk, *CM* camel milk, *DM* donkey milk, *MM* mare milk, *HM* human milk. The notes for Table 2 are the same as those for Table 1.

**Table 2:** Intrapositional distribution of fatty acids in milk fat triacylglycerols across eight mammalian species and human milk

Fatty acid	Intrapositional sn-2 (mol%)										Intrapositional sn-1/3 (mol%)									
	BOM	BUM	YM	GM	SM	CM	DM	MM	HM	*P*-value	BOM	BUM	YM	GM	SM	CM	DM	MM	HM	*P*-value
SFA																				
ECSFA																				
C4:0	0.90	1.08	1.18	0.49	0.94	0.01	0.11	0.09	0.02	<0.01	12.74	26.36	22.42	8.87	16.41	0.18	0.77	0.47	0.09	<0.01
	(0.06)	(0.06)	(0.19)	(0.04)	(0.05)	(0.003)	(0.02)	(0.01)	(0.01)		(1.37)	(1.55)	(3.23)	(0.36)	(1.57)	(0.01)	(0.13)	(0.08)	(0.003)	
C6:0	0.74	0.64	1.10	0.82	0.86	0.04	0.27	0.18	0.02	<0.01	6.20	7.30	10.87	5.93	9.57	0.74	1.01	0.63	0.11	<0.01
	(0.03)	(0.003)	(0.05)	(0.05)	(0.11)	(0.004)	(0.09)	(0.02)	(0.01)		(0.66)	(0.63)	(2.61)	(0.25)	(0.79)	(0.02)	(0.23)	(0.02)	(0.01)	
C8:0	1.19	0.85	1.31	2.78	2.20	0.13	4.01	2.78	0.08	<0.01	1.96	0.72	1.69	2.74	2.61	0.21	4.92	4.19	0.29	<0.01
	(0.06)	(0.04)	(0.28)	(0.20)	(0.33)	(0.01)	(0.67)	(0.23)	(0.01)		(0.02)	(0.1)	(0.24)	(0.2)	(0.51)	(0.02)	(0.14)	(0.6)	(0.02)	
C10:0	2.63	1.48	2.26	10.32	6.38	0.18	6.55	6.52	0.89	<0.01	3.34	1.21	2.20	10.24	6.29	0.23	6.84	6.91	1.77	<0.01
	(0.11)	(0.07)	(0.52)	(0.77)	(0.84)	(0.02)	(0.91)	(0.39)	(0.07)		(0.04)	(0.15)	(0.34)	(0.74)	(0.90)	(0.01)	(0.26)	(0.71)	(0.10)	
C12:0	3.48	2.59	2.28	6.91	4.25	1.37	7.20	9.17	4.61	<0.01	3.03	1.00	1.33	4.24	2.98	1.09	3.26	5.56	3.57	<0.01
	(0.10)	(0.19)	(0.55)	(0.62)	(0.27)	(0.02)	(0.21)	(0.64)	(0.45)		(0.07)	(0.22)	(0.25)	(0.38)	(0.27)	(0.06)	(0.44)	(0.75)	(0.35)	
C14:0	15.78	15.01	10.37	17.94	17.11	21.80	6.32	12.34	5.38	<0.01	6.89	2.14	4.76	5.32	5.80	11.97	1.66	4.81	2.86	<0.01
	(0.35)	(1.02)	(1.23)	(1.75)	(0.39)	(1.03)	(0.02)	(0.54)	(0.31)		(0.50)	(0.61)	(0.45)	(0.30)	(0.49)	(0.79)	(0.18)	(0.39)	(0.19)	
C16:0	39.13	34.69	34.81	29.66	23.76	26.57	26.72	32.18	36.53	<0.01	27.05	22.18	18.64	25.70	23.16	26.39	14.92	20.26	14.80	<0.01
	(1.08)	(1.65)	(2.28)	(1.30)	(0.25)	(1.54)	(2.82)	(0.22)	(0.61)		(1.76)	(2.88)	(2.21)	(0.99)	(0.74)	(0.80)	(1.33)	(0.21)	(1.57)	
C18:0	5.40	9.03	9.51	2.97	8.41	7.76	0.94	0.68	3.13	<0.01	8.87	12.28	14.18	4.92	9.56	21.30	0.60	0.48	7.44	<0.01
	(0.22)	(0.88)	(0.43)	(0.09)	(0.61)	(0.47)	(0.03)	(0.06)	(0.41)		(0.37)	(0.91)	(1.24)	(0.13)	(1.11)	(1.30)	(0.06)	(0.06)	(1.31)	
C20:0	0.04	0.08	0.22	0.10	0.27	0.08	0.08	0.04	NA	<0.01	0.04	0.20	0.25	0.17	0.17	0.23	NA	0.03	0.11	<0.01
	(0.003)	(0.01)	(0.03)	(0.004)	(0.05)	(0.01)	(0.01)	(0.001)	NA		(0.004)	(0.03)	(0.07)	(0.01)	(0.05)	(0.04)	NA	(0.004)	(0.001)	
C22:0	0.02	0.05	0.08	0.04	0.08	0.01	0.13	NA	0.06	<0.01	0.01	0.06	0.04	0.19	0.01	0.10	NA	NA	0.01	<0.01
	(0.0004)	(0.003)	(0.01)	(0.01)	(0.0003)	(0.001)	(0.05)	NA	(0.003)		(0.001)	(0.01)	(0.01)	(0.01)	(0.002)	(0.02)	NA	NA	(0.001)	
C24:0	0.01	NA	0.04	0.02	0.03	0.01	NA	NA	0.05	<0.01	0.01	NA	0.02	0.10	0.01	0.03	NA	NA	0.001	<0.01
	(0.002)	NA	(0.01)	(0.001)	(0.004)	(0.001)	NA	NA	(0.01)		(0.0003)	NA	(0.004)	(0.01)	(0.001)	(0.0002)	NA	NA	(0.001)	
OCSFA																				
C5:0	NA	NA	NA	NA	NA	NA	NA	NA	NA	<0.01	0.11	0.18	0.06	0.05	0.09	NA	NA	NA	NA	<0.01
	NA	NA	NA	NA	NA	NA	NA	NA	NA		(0.02)	(0.0001)	(0.01)	(0.004)	(0.02)	NA	NA	NA	NA	
C7:0	0.01	0.003	0.003	0.01	0.01	NA	NA	0.01	NA	<0.01	0.06	0.02	0.02	0.04	0.04	NA	NA	0.02	NA	<0.01
	(0.0001)	(0.0001)	(0.0004)	(0.001)	(0.001)	NA	NA	(0.001)	NA		(0.0001)	(0.003)	(0.003)	(0.001)	(0.01)	NA	NA	(0.002)	NA	
C9:0	0.03	0.01	0.01	0.06	0.04	0.01	0.04	0.01	0.01	<0.01	0.05	0.02	0.01	0.06	0.05	0.01	0.04	0.02	0.01	<0.01
	(0.001)	(0.01)	(0.001)	(0.01)	(0.01)	(0.001)	(0.003)	(0.001)	(0.0001)		(0.003)	(0.001)	(0.001)	(0.001)	(0.01)	(0.001)	(0.002)	(0.0001)	(0.001)	
C11:0	0.07	0.02	0.02	0.12	0.07	0.01	0.05	NA	0.02	<0.01	0.07	0.02	0.01	0.03	0.05	0.04	0.02	0.02	0.01	<0.01
	(0.001)	(0.001)	(0.003)	(0.02)	(0.01)	(0.0001)	(0.01)	NA	(0.0001)		(0.003)	(0.001)	(0.002)	(0.003)	(0.01)	(0.001)	(0.002)	(0.004)	(0.001)	
C13:0	0.14	0.07	0.10	0.15	0.13	0.12	NA	0.09	0.03	<0.01	0.07	0.06	0.03	0.06	0.06	0.08	0.03	NA	0.02	<0.01
	(0.01)	(0.01)	(0.01)	(0.01)	(0.01)	(0.02)	NA	(0.01)	(0.0001)		(0.01)	(0.01)	(0.01)	(0.001)	(0.01)	(0.01)	(0.004)	NA	(0.002)	
C15:0	0.96	1.03	1.78	0.78	1.00	1.58	0.36	0.53	0.26	<0.01	0.68	0.62	0.72	0.39	0.68	0.94	0.05	NA	0.10	<0.01
	(0.01)	(0.04)	(0.13)	(0.08)	(0.02)	(0.05)	(0.01)	(0.01)	(0.03)		(0.02)	(0.03)	(0.09)	(0.04)	(0.04)	(0.05)	(0.01)	NA	(0.03)	
C17:0	0.43	0.51	1.04	0.47	0.65	0.50	0.42	0.28	0.29	<0.01	0.37	0.23	0.51	0.26	0.42	0.49	NA	NA	0.11	<0.01
	(0.01)	(0.02)	(0.06)	(0.06)	(0.02)	(0.04)	(0.02)	(0.02)	(0.04)		(0.02)	(0.02)	(0.10)	(0.01)	(0.03)	(0.02)	NA	NA	(0.02)	
C21:0	0.02	0.03	0.04	0.03	0.04	0.01	NA	NA	NA	<0.01	NA	0.01	0.02	0.22	0.01	0.16	NA	NA	0.004	<0.01
	(0.01)	(0.002)	(0.01)	(0.003)	(0.003)	(0.002)	NA	NA	NA		NA	(0.002)	(0.01)	(0.02)	(0.0004)	(0.01)	NA	NA	(0.001)	
C23:0	0.01	0.02	0.03	0.01	0.03	0.003	NA	NA	NA	<0.01	0.01	0.06	0.02	0.11	0.01	0.06	NA	NA	0.003	<0.01
	(0.0004)	(0.0005)	(0.003)	(0.002)	(0.001)	(0.001)	NA	NA	NA		(0.001)	(0.005)	(0.01)	(0.01)	(0.002)	(0.01)	NA	NA	(0.0003)	
BCSFA																				
iso C13:0	0.03	0.04	0.08	0.04	0.04	0.07	NA	0.05	NA	<0.01	0.005	0.01	0.01	0.03	0.02	0.04	0.01	NA	0.002	<0.01
	(0.002)	(0.01)	(0.001)	(0.005)	(0.001)	(0.01)	NA	(0.003)	NA		(0.001)	(0.0004)	(0.003)	(0.002)	(0.001)	(0.003)	(0.001)	NA	(0.0005)	
anteiso C13:0	0.01	0.02	0.02	0.01	0.01	0.03	NA	NA	NA	<0.01	0.002	0.001	0.003	0.01	0.005	0.01	NA	NA	NA	<0.01
	(0.001)	(0.003)	(0.003)	(0.001)	(0.001)	(0.004)	NA	NA	NA		(0.0001)	(0.0004)	(0.001)	(0.001)	(0.001)	(0.0002)	NA	NA	NA	
iso C14:0	0.07	0.15	0.27	0.09	0.20	0.16	NA	0.13	NA	<0.01	0.04	0.02	0.11	0.06	0.07	0.13	0.02	0.004	0.01	<0.01
	(0.004)	(0.01)	(0.03)	(0.005)	(0.02)	(0.02)	NA	(0.01)	NA		(0.001)	(0.003)	(0.02)	(0.001)	(0.01)	(0.01)	(0.0002)	(0.003)	(0.0001)	
iso C15:0	0.14	0.21	0.35	0.13	0.28	0.30	NA	NA	NA	<0.01	0.08	0.11	0.18	0.06	0.14	0.28	0.01	0.02	0.02	<0.01
	(0.01)	(0.02)	(0.003)	(0.01)	(0.01)	(0.03)	NA	NA	NA		(0.003)	(0.02)	(0.02)	(0.004)	(0.02)	(0.01)	(0.001)	(0.002)	(0.01)	
anteiso C15:0	0.25	0.35	0.57	0.22	0.47	0.63	NA	NA	0.05	<0.01	0.20	0.22	0.35	0.09	0.28	0.64	0.02	NA	0.02	<0.01
	(0.02)	(0.01)	(0.05)	(0.03)	(0.01)	(0.06)	NA	NA	(0.02)		(0.01)	(0.02)	(0.04)	(0.02)	(0.02)	(0.03)	(0.004)	NA	(0.01)	
iso C16:0	0.15	0.18	0.32	0.16	0.32	0.29	0.18	0.29	NA	<0.01	0.10	0.12	0.22	0.19	0.23	0.30	NA	0.004	0.03	<0.01
	(0.004)	(0.02)	(0.02)	(0.01)	(0.04)	(0.02)	(0.01)	(0.02)	NA		(0.01)	(0.02)	(0.03)	(0.003)	(0.02)	(0.01)	NA	(0.002)	(0.01)	
iso C17:0	0.11	0.09	0.19	0.09	0.14	0.17	NA	0.04	NA	<0.01	0.15	0.13	0.21	0.13	0.16	0.31	NA	NA	0.03	<0.01
	(0.002)	(0.005)	(0.02)	(0.01)	(0.001)	(0.02)	NA	(0.01)	NA		(0.004)	(0.01)	(0.03)	(0.01)	(0.03)	(0.02)	NA	NA	(0.01)	
anteiso C17:0	0.22	0.20	0.36	0.16	0.31	0.60	0.27	0.19	0.12	<0.01	0.24	0.17	0.36	0.21	0.34	0.53	NA	0.09	0.03	<0.01
	(0.01)	(0.003)	(0.03)	(0.02)	(0.01)	(0.03)	(0.10)	(0.01)	(0.05)		(0.001)	(0.02)	(0.06)	(0.02)	(0.01)	(0.03)	NA	(0.02)	(0.01)	
iso C18:0	NA	NA	NA	NA	NA	NA	NA	NA	NA	/	0.02	0.03	0.05	0.08	0.05	0.10	0.01	0.03	NA	<0.01
	NA	NA	NA	NA	NA	NA	NA	NA	NA		(0.002)	(0.01)	(0.01)	(0.0001)	(0.01)	(0.003)	(0.001)	(0.001)	NA	
UFA																				
MUFA																				
C10:1 c9	0.25	0.02	0.17	0.40	0.24	0.02	0.57	1.41	NA	<0.01	0.26	0.005	0.12	0.16	0.16	0.04	1.15	1.09	0.01	<0.01
	(0.02)	(0.002)	(0.03)	(0.04)	(0.01)	(0.01)	(0.10)	(0.27)	NA		(0.001)	(0.001)	(0.02)	(0.01)	(0.01)	(0.001)	(0.05)	(0.37)	(0.0002)	
C14:1 c9	0.94	0.52	0.27	0.34	0.18	0.77	NA	1.11	NA	<0.01	0.82	0.18	0.12	0.18	0.11	0.49	0.14	NA	0.10	<0.01
	(0.04)	(0.04)	(0.04)	(0.02)	(0.02)	(0.13)	NA	(0.22)	NA		(0.04)	(0.02)	(0.03)	(0.02)	(0.03)	(0.09)	(0.01)	NA	(0.02)	
C16:1 t9	NA	0.13	0.11	0.07	NA	NA	NA	NA	NA	<0.01	NA	0.13	0.07	0.13	0.08	0.24	NA	NA	NA	<0.01
	NA	(0.01)	(0.01)	(0.01)	NA	NA	NA	NA	NA		NA	(0.02)	(0.01)	(0.02)	(0.02)	(0.04)	NA	NA	NA	
C16:1 c7	0.09	0.10	0.16	0.12	0.17	0.28	0.73	0.24	0.24	<0.01	0.17	0.24	0.24	0.29	0.25	0.61	0.52	0.19	0.21	<0.01
	(0.001)	(0.01)	(0.03)	(0.01)	(0.01)	(0.04)	(0.01)	(0.01)	(0.01)		(0.01)	(0.01)	(0.03)	(0.01)	(0.05)	(0.05)	(0.02)	(0.02)	(0.03)	
C16:1 c9	1.65	1.10	1.01	0.72	0.84	6.07	2.38	4.34	1.19	<0.01	1.90	0.74	0.88	0.73	0.69	4.22	2.15	5.35	1.23	<0.01
	(0.06)	(0.11)	(0.23)	(0.06)	(0.03)	(0.56)	(0.04)	(0.21)	(0.09)		(0.10)	(0.06)	(0.24)	(0.04)	(0.08)	(0.61)	(0.04)	(0.47)	(0.12)	
C17:1 c10	NA	NA	NA	NA	NA	NA	NA	NA	NA	/	NA	0.16	NA	0.28	NA	0.46	NA	NA	NA	<0.01
	NA	NA	NA	NA	NA	NA	NA	NA	NA		NA	(0.02)	NA	(0.02)	NA	(0.03)	NA	NA	NA	
C18:1 t6	0.17	0.19	0.15	0.09	0.15	0.63	NA	NA	NA	<0.01	0.17	0.36	0.11	0.29	0.17	0.53	0.01	NA	0.04	<0.01
	(0.01)	(0.03)	(0.02)	(0.01)	(0.02)	(0.04)	NA	NA	NA		(0.03)	(0.02)	(0.004)	(0.01)	(0.05)	(0.03)	(0.001)	NA	(0.01)	
C18:1 t9	0.08	NA	0.09	0.09	0.12	NA	NA	NA	NA	<0.01	NA	0.23	0.13	0.28	0.13	0.34	0.04	NA	0.05	<0.01
	(0.01)	NA	(0.01)	(0.01)	(0.01)	NA	NA	NA	NA		NA	(0.02)	(0.01)	(0.02)	(0.02)	(0.03)	(0.02)	NA	(0.01)	
C18:1 t11	0.28	2.35	1.28	0.32	0.51	0.60	NA	NA	0.06	<0.01	0.51	3.33	2.11	0.50	0.58	1.67	0.01	NA	0.10	<0.01
	(0.01)	(0.26)	(0.19)	(0.06)	(0.08)	(0.23)	NA	NA	(0.03)		(0.01)	(0.38)	(0.41)	(0.11)	(0.10)	(0.59)	(0.002)	NA	(0.05)	
C18:1 c6	0.07	0.11	0.06	0.06	0.11	0.08	NA	NA	NA	<0.01	0.27	0.28	0.06	0.20	0.15	0.53	0.01	NA	0.05	<0.01
	(0.004)	(0.01)	(0.01)	(0.001)	(0.01)	(0.01)	NA	NA	NA		(0.02)	(0.03)	(0.02)	(0.01)	(0.03)	(0.05)	(0.001)	NA	(0.01)	
C18:1 c9	20.42	23.93	24.98	19.30	26.74	24.43	19.26	11.78	24.21	<0.01	19.04	14.44	13.77	18.20	15.77	17.27	31.96	22.06	30.85	<0.01
	(1.19)	(1.70)	(4.42)	(2.76)	(2.19)	(1.67)	(0.55)	(0.57)	(1.42)		(0.26)	(0.26)	(1.86)	(0.36)	(0.61)	(1.30)	(0.45)	(0.57)	(1.62)	
C18:1 c11	0.34	0.09	0.29	0.17	0.20	0.27	0.53	0.27	0.82	<0.01	1.23	1.01	0.45	1.25	0.63	1.50	0.73	0.83	1.49	<0.01
	(0.02)	(0.002)	(0.04)	(0.05)	(0.07)	(0.05)	(0.02)	(0.01)	(0.09)		(0.10)	(0.05)	(0.17)	(0.06)	(0.36)	(0.23)	(0.1)	(0.07)	(0.13)	
C18:1 c12	0.13	0.12	0.06	0.10	0.15	0.11	NA	NA	NA	<0.01	0.26	0.14	0.06	0.10	0.16	0.25	0.06	NA	0.17	<0.01
	(0.01)	(0.01)	(0.02)	(0.001)	(0.02)	(0.01)	NA	NA	NA		(0.01)	(0.03)	(0.03)	(0.01)	(0.03)	(0.01)	(0.01)	NA	(0.01)	
C20:1 t11	NA	NA	NA	NA	NA	NA	NA	NA	NA	/	NA	NA	NA	NA	0.01	NA	NA	NA	NA	<0.01
	NA	NA	NA	NA	NA	NA	NA	NA	NA		NA	NA	NA	NA	(0.001)	NA	NA	NA	NA	
C20:1 c8	0.08	0.09	0.16	NA	NA	NA	NA	NA	NA	<0.01	0.03	0.06	0.08	NA	NA	0.10	NA	NA	NA	<0.01
	(0.01)	(0.01)	(0.01)	NA	NA	NA	NA	NA	NA		(0.004)	(0.004)	(0.02)	NA	NA	(0.003)	NA	NA	NA	
C20:1 c11	NA	NA	NA	0.05	0.05	0.09	0.25	0.38	0.28	<0.01	NA	NA	NA	0.15	0.02	0.11	0.14	0.60	0.13	<0.01
	NA	NA	NA	(0.003)	(0.001)	(0.01)	(0.01)	(0.08)	(0.01)		NA	NA	NA	(0.01)	(0.002)	(0.003)	(0.01)	(0.10)	(0.01)	
C22:1 t13	NA	NA	NA	NA	NA	NA	NA	NA	NA	/	NA	NA	NA	NA	NA	NA	NA	NA	NA	/
	NA	NA	NA	NA	NA	NA	NA	NA	NA		NA	NA	NA	NA	NA	NA	NA	NA	NA	
C22:1 c13	NA	NA	NA	NA	NA	NA	NA	NA	NA	/	NA	NA	NA	0.27	NA	0.12	NA	NA	NA	<0.01
	NA	NA	NA	NA	NA	NA	NA	NA	NA		NA	NA	NA	(0.03)	NA	(0.002)	NA	NA	NA	
C24:1 c15	NA	NA	NA	NA	NA	NA	NA	NA	NA	/	NA	NA	0.01	NA	0.01	NA	0.09	NA	0.01	<0.01
	NA	NA	NA	NA	NA	NA	NA	NA	NA		NA	NA	(0.001)	NA	(0.001)	NA	(0.07)	NA	(0.002)	
PUFA																				
C18:2 t9t12	NA	NA	NA	NA	NA	NA	NA	NA	NA	/	NA	NA	NA	0.30	NA	0.17	NA	NA	NA	<0.01
	NA	NA	NA	NA	NA	NA	NA	NA	NA		NA	NA	NA	(0.02)	NA	(0.01)	NA	NA	NA	
C18:2 c9t12	NA	NA	NA	NA	NA	NA	NA	NA	NA	/	0.04	0.15	0.07	0.09	0.07	0.10	0.02	0.04	0.06	<0.01
	NA	NA	NA	NA	NA	NA	NA	NA	NA		(0.003)	(0.04)	(0.05)	(0.01)	(0.02)	(0.01)	(0.004)	(0.001)	(0.001)	
C18:2 t9c12	NA	NA	NA	NA	NA	0.03	NA	NA	NA	<0.01	NA	0.20	NA	NA	0.03	0.11	NA	NA	0.04	<0.01
	NA	NA	NA	NA	NA	(0.01)	NA	NA	NA		NA	(0.03)	NA	NA	(0.005)	(0.02)	NA	NA	(0.01)	
C18:2 c9c12	2.29	1.25	1.17	2.71	1.63	2.43	21.09	4.80	17.95	<0.01	2.00	0.95	1.00	2.00	1.04	1.03	27.96	4.74	30.65	<0.01
	(0.10)	(0.14)	(0.16)	(1.06)	(0.12)	(0.57)	(1.44)	(0.23)	(2.33)		(0.13)	(0.18)	(0.24)	(0.77)	(0.10)	(0.16)	(1.60)	(0.27)	(4.16)	
C18:3 c6c9c12	0.02	0.01	0.004	0.02	0.05	0.01	NA	NA	0.03	<0.01	0.02	0.05	0.003	0.27	0.03	0.19	0.01	0.10	0.07	<0.01
	(0.001)	(0.001)	(0.001)	(0.01)	(0.03)	(0.002)	NA	NA	(0.01)		(0.002)	(0.01)	(0.0001)	(0.02)	(0.01)	(0.01)	(0.001)	(0.03)	(0.004)	
C18:3 c9c12c15	0.19	0.11	0.63	0.17	0.13	0.72	1.23	9.36	0.66	<0.01	0.20	0.19	0.56	0.36	0.15	0.54	0.63	20.45	2.36	<0.01
	(0.01)	(0.002)	(0.13)	(0.08)	(0.06)	(0.09)	(0.07)	(1.59)	(0.07)		(0.03)	(0.06)	(0.16)	(0.05)	(0.07)	(0.05)	(0.04)	(2.79)	(0.27)	
C18:2 c9t11	0.21	1.06	0.55	0.44	0.33	0.46	NA	NA	NA	<0.01	0.25	0.86	0.67	0.23	0.23	0.30	0.05	0.05	0.07	<0.01
	(0.01)	(0.04)	(0.09)	(0.10)	(0.03)	(0.11)	NA	NA	NA		(0.04)	(0.12)	(0.20)	(0.02)	(0.03)	(0.03)	(0.003)	(0.002)	(0.02)	
C18:2 c9c11	NA	0.12	0.17	NA	NA	0.12	NA	NA	NA	<0.01	NA	0.32	NA	NA	NA	0.30	NA	NA	NA	<0.01
	NA	(0.02)	(0.02)	NA	NA	(0.06)	NA	NA	NA		NA	(0.04)	NA	NA	NA	(0.06)	NA	NA	NA	
C20:2 c11c14	0.02	NA	0.02	NA	0.01	0.01	0.32	NA	0.21	<0.01	0.01	NA	0.0002	NA	0.003	0.07	0.10	NA	0.07	<0.01
	(0.0001)	NA	(0.003)	NA	(0.001)	(0.002)	(0.04)	NA	(0.02)		(0.001)	NA	(0.0001)	NA	(0.001)	(0.003)	(0.01)	NA	(0.004)	
C20:3 c8c11c14	0.73	0.22	0.16	0.12	0.21	0.20	NA	0.62	2.15	<0.01	0.40	0.41	0.05	2.07	0.06	1.49	0.15	0.98	0.43	<0.01
	(0.01)	(0.01)	(0.04)	(0.04)	(0.02)	(0.03)	NA	(0.10)	(0.16)		(0.02)	(0.08)	(0.01)	(0.11)	(0.01)	(0.04)	(0.004)	(0.10)	(0.05)	
C20:3 c11c14c17	NA	NA	0.01	NA	NA	NA	NA	0.04	NA	<0.01	NA	0.05	0.02	NA	NA	0.06	0.06	0.09	0.02	<0.01
	NA	NA	(0.002)	NA	NA	NA	NA	(0.0003)	NA		NA	(0.0001)	(0.01)	NA	NA	(0.0001)	(0.02)	(0.01)	(0.004)	
C20:4 c5c8c11c14	0.05	0.03	0.04	0.05	0.08	0.06	NA	0.04	0.28	<0.01	0.06	0.05	0.05	0.37	0.07	0.26	0.04	0.04	0.24	<0.01
	(0.002)	(0.001)	(0.004)	(0.004)	(0.005)	(0.01)	NA	(0.004)	(0.01)		(0.01)	(0.01)	(0.01)	(0.02)	(0.01)	(0.01)	(0.002)	(0.003)	(0.02)	
C22:2 c13c16	NA	NA	NA	NA	NA	NA	NA	NA	NA	/	NA	NA	NA	NA	NA	NA	NA	NA	0.01	<0.01
	NA	NA	NA	NA	NA	NA	NA	NA	NA		NA	NA	NA	NA	NA	NA	NA	NA	(0.001)	
C20:5 c5c8c11c14c17	0.02	NA	0.02	0.02	NA	0.05	NA	NA	0.04	<0.01	0.01	NA	0.01	0.21	0.01	0.09	NA	NA	0.01	<0.01
	(0.001)	NA	(0.01)	(0.004)	NA	(0.01)	NA	NA	(0.01)		(0.0001)	NA	(0.01)	(0.01)	(0.0001)	(0.03)	NA	NA	(0.004)	
C22:3 c13c16c19	NA	NA	NA	NA	NA	NA	NA	NA	NA	/	NA	NA	NA	NA	NA	NA	NA	0.04	NA	<0.01
	NA	NA	NA	NA	NA	NA	NA	NA	NA		NA	NA	NA	NA	NA	NA	NA	(0.004)	NA	
C22:4 c7c10c13c16	0.02	NA	0.01	0.02	0.03	0.04	NA	NA	0.10	<0.01	0.003	NA	0.001	0.19	0.003	0.13	NA	NA	0.004	<0.01
	(0.001)	NA	(0.002)	(0.004)	(0.003)	(0.01)	NA	NA	(0.01)		(0.001)	NA	(0.0001)	(0.01)	(0.001)	(0.0001)	NA	NA	(0.002)	
C22:5 c4c7c10c13c16	NA	NA	0.01	0.01	0.01	0.01	NA	NA	0.07	<0.01	0.003	NA	NA	0.19	0.002	NA	NA	NA	0.01	<0.01
	NA	NA	(0.002)	(0.004)	(0.001)	(0.001)	NA	NA	(0.01)		(0.0002)	NA	NA	(0.01)	(0.0004)	NA	NA	NA	(0.001)	
C22:5 c7c10c13c16c19	0.02	0.02	0.05	0.03	0.02	0.08	NA	NA	0.05	<0.01	0.001	0.003	0.02	0.10	0.001	0.05	NA	NA	0.01	<0.01
	(0.0004)	(0.003)	(0.01)	(0.004)	(0.002)	(0.01)	NA	NA	(0.002)		(0.0002)	(0.0002)	(0.01)	(0.01)	(0.0003)	(0.01)	NA	NA	(0.001)	
C22:6 c4c7c10c13c16c19	NA	NA	0.01	0.01	0.01	NA	NA	0.03	0.13	<0.01	NA	NA	0.002	0.12	0.0003	NA	NA	NA	0.04	<0.01
	NA	NA	(0.002)	(0.001)	(0.001)	NA	NA	(0.01)	(0.02)		NA	NA	(0.0004)	(0.01)	(0.0002)	NA	NA	NA	(0.01)	
FA group																				
SFA	71.96	68.45	68.35	74.59	68.02	62.44	53.66	65.58	51.53	<0.01	72.39	75.48	79.42	70.50	79.38	66.60	34.19	43.55	31.46	<0.01
	(1.36)	(1.96)	(4.88)	(3.48)	(2.21)	(2.13)	(1.14)	(1.64)	(1.05)		(0.70)	(0.81)	(3.26)	(1.14)	(0.22)	(1.44)	(2.08)	(2.60)	(2.59)	
ECSFA	69.31	65.49	63.16	72.05	64.29	57.95	52.32	63.97	50.76	<0.01	70.15	73.45	76.39	68.41	76.59	62.48	33.98	43.34	31.06	<0.01
	(1.33)	(1.97)	(5.02)	(3.27)	(2.13)	(2.01)	(1.23)	(1.59)	(1.07)		(0.78)	(0.80)	(3.63)	(1.11)	(0.36)	(1.40)	(2.07)	(2.58)	(2.51)	
OCSFA	1.67	1.71	3.02	1.63	1.96	2.24	0.88	0.92	0.60	<0.01	1.41	1.22	1.41	1.23	1.43	1.78	0.13	0.05	0.25	<0.01
	(0.02)	(0.06)	(0.19)	(0.18)	(0.02)	(0.04)	(0.02)	(0.02)	(0.08)		(0.06)	(0.05)	(0.19)	(0.05)	(0.08)	(0.05)	(0.01)	(0.01)	(0.05)	
BCSFA	0.98	1.24	2.17	0.91	1.77	2.24	0.46	0.69	0.17	<0.01	0.83	0.81	1.62	0.86	1.36	2.34	0.08	0.16	0.15	<0.01
	(0.02)	(0.03)	(0.03)	(0.07)	(0.09)	(0.14)	(0.11)	(0.03)	(0.06)		(0.02)	(0.08)	(0.19)	(0.04)	(0.08)	(0.08)	(0.004)	(0.01)	(0.06)	
UFA	28.04	31.55	31.65	25.41	31.98	37.56	46.34	34.42	48.47	<0.01	27.65	24.52	20.67	29.50	20.62	33.40	66.03	56.64	68.55	<0.01
	(1.36)	(1.96)	(4.88)	(3.48)	(2.21)	(2.13)	(1.14)	(1.64)	(1.05)		(0.70)	(0.81)	(3.26)	(1.14)	(0.22)	(1.44)	(2.03)	(2.56)	(2.59)	
n-1 UFA	0.25	0.02	0.17	0.40	0.24	0.02	0.57	1.41	NA	<0.01	0.26	0.005	0.12	0.16	0.16	0.04	1.15	1.09	0.01	<0.01
	(0.02)	(0.002)	(0.03)	(0.04)	(0.01)	(0.002)	(0.10)	(0.27)	NA		(0.005)	(0.001)	(0.02)	(0.01)	(0.01)	(0.003)	(0.05)	(0.37)	(0.001)	
n-3 UFA	0.22	0.13	0.72	0.23	0.17	0.85	1.23	9.43	0.88	<0.01	0.20	0.24	0.62	0.77	0.16	0.75	0.69	20.57	2.45	<0.01
	(0.01)	(0.001)	(0.15)	(0.07)	(0.05)	(0.09)	(0.07)	(1.58)	(0.08)		(0.03)	(0.07)	(0.18)	(0.07)	(0.07)	(0.08)	(0.04)	(2.80)	(0.26)	
n-5 UFA	0.94	0.52	0.27	0.34	0.18	0.77	NA	1.11	NA	<0.01	0.82	0.18	0.12	0.18	0.11	0.49	0.14	NA	0.10	<0.01
	(0.04)	(0.04)	(0.04)	(0.02)	(0.02)	(0.13)	NA	(0.22)	NA		(0.04)	(0.02)	(0.03)	(0.02)	(0.03)	(0.09)	(0.01)	NA	(0.02)	
n-6 UFA	3.26	1.63	1.47	3.03	2.17	2.89	21.4	5.46	20.79	<0.01	2.79	1.94	1.23	5.59	1.46	3.82	28.35	5.90	31.77	<0.01
	(0.10)	(0.13)	(0.22)	(1.10)	(0.08)	(0.63)	(1.46)	(0.13)	(2.35)		(0.17)	(0.26)	(0.33)	(0.97)	(0.14)	(0.16)	(1.60)	(0.16)	(4.13)	
n-7 UFA	2.47	4.84	3.42	1.71	1.88	7.52	2.91	4.60	2.07	<0.01	3.89	6.56	4.18	3.12	2.21	8.69	2.94	6.23	2.89	<0.01
	(0.09)	(0.21)	(0.42)	(0.08)	(0.01)	(0.36)	(0.02)	(0.22)	(0.14)		(0.19)	(0.43)	(0.96)	(0.15)	(0.45)	(0.11)	(0.13)	(0.47)	(0.17)	
n-9 UFA	20.58	24.03	25.23	19.56	27.08	24.80	20.23	12.41	24.73	<0.01	19.21	14.90	14.14	19.19	16.19	18.45	32.74	22.85	31.25	<0.01
	(1.20)	(1.71)	(4.45)	(2.75)	(2.18)	(1.69)	(0.54)	(0.63)	(1.42)		(0.27)	(0.26)	(1.89)	(0.36)	(0.56)	(1.34)	(0.38)	(0.67)	(1.65)	
n-12 UFA	0.32	0.39	0.36	0.14	0.27	0.71	NA	NA	NA	<0.01	0.46	0.69	0.25	0.49	0.32	1.15	0.02	NA	0.09	<0.01
	(0.02)	(0.04)	(0.01)	(0.02)	(0.02)	(0.05)	NA	NA	NA		(0.04)	(0.05)	(0.04)	(0.03)	(0.08)	(0.04)	(0.002)	NA	(0.02)	
MUFA	24.49	28.74	28.78	21.81	29.46	33.35	23.71	19.53	26.80	<0.01	24.67	21.31	18.20	23.02	18.93	28.48	37.00	30.12	34.44	<0.01
	(1.25)	(1.88)	(4.66)	(2.67)	(2.21)	(2.25)	(0.45)	(1.06)	(1.53)		(0.49)	(0.58)	(2.68)	(0.37)	(0.09)	(1.60)	(0.45)	(0.39)	(1.83)	
PUFA	3.56	2.81	2.86	3.60	2.52	4.21	22.63	14.89	21.67	<0.01	2.98	3.21	2.46	6.49	1.69	4.92	29.03	26.52	34.12	<0.01
	(0.11)	(0.17)	(0.29)	(1.25)	(0.09)	(0.74)	(1.54)	(1.57)	(2.43)		(0.22)	(0.31)	(0.62)	(1.05)	(0.17)	(0.17)	(1.59)	(2.64)	(4.39)	
SCFA	1.63	1.72	2.28	1.32	1.80	0.05	0.38	0.26	0.04	<0.01	19.05	33.83	33.48	14.86	26.15	0.92	1.78	1.10	0.20	<0.01
	(0.09)	(0.07)	(0.23)	(0.08)	(0.15)	(0.01)	(0.11)	(0.03)	(0.01)		(2.04)	(2.12)	(5.84)	(0.61)	(2.37)	(0.01)	(0.37)	(0.09)	(0.02)	
MCFA	23.68	20.26	16.89	38.83	30.67	23.89	24.74	32.50	11.01	<0.01	15.78	5.22	10.28	22.99	18.15	13.86	17.94	22.62	8.55	<0.01
	(0.62)	(1.18)	(2.42)	(3.39)	(1.68)	(1.11)	(1.63)	(1.92)	(0.77)		(0.56)	(0.57)	(1.27)	(1.57)	(2.03)	(0.90)	(0.74)	(2.81)	(0.28)	
LCFA	74.69	78.02	80.83	59.86	67.52	76.06	74.88	67.23	88.94	<0.01	65.16	60.95	56.24	62.15	55.70	85.22	80.28	76.28	91.25	<0.01
	(0.71)	(1.23)	(2.65)	(3.43)	(1.84)	(1.11)	(1.72)	(1.94)	(0.76)		(1.48)	(1.83)	(7.11)	(2.00)	(0.98)	(0.91)	(1.10)	(2.89)	(0.27)

**Table 3:** Interpositional distribution of fatty acids in milk fat triacylglycerols across eight mammalian species and human milk

Fatty acid	Interpositional sn-2 (mol%)									Interpositional sn-1/3 (mol%)								
	BOM	BUM	YM	GM	SM	CM	DM	MM	HM	BOM	BUM	YM	GM	SM	CM	DM	MM	HM
SFA																		
ECSFA																		
C4:0	3.53^*^	2.00	2.56^*^	2.74^*^	2.85^*^	2.78^*^	6.64^*^	8.92^*^	9.99^*^	48.24^*^	49.00	48.72^*^	48.63^*^	48.58^*^	48.61^*^	46.68^*^	45.54^*^	45.00
	(0.62)	(0.01)	(0.14)	(0.32)	(0.37)	(0.91)	(1.37)	(1.23)	(2.27)	(0.31)	(0.01)	(0.07)	(0.16)	(0.19)	(0.46)	(0.68)	(0.61)	(1.14)
C6:0	5.76^*^	4.28^*^	5.42^*^	6.51^*^	4.42^*^	2.32^*^	12.08^*^	12.23^*^	9.29^*^	47.12^*^	47.86^*^	47.29^*^	46.75^*^	47.79^*^	48.84^*^	43.96^*^	43.89^*^	45.36^*^
	(0.85)	(0.36)	(1.31)	(0.56)	(0.79)	(0.22)	(3.74)	(1.60)	(3.32)	(0.43)	(0.18)	(0.65)	(0.28)	(0.39)	(0.11)	(1.87)	(0.80)	(1.66)
C8:0	23.26^*^	37.47^*^	27.40^*^	33.70^*^	30.11^*^	23.70^*^	28.70^*^	25.23^*^	12.57^*^	38.37^*^	31.26^*^	36.30^*^	33.15	34.95^*^	38.15^*^	35.65^*^	37.39^*^	43.72^*^
	(0.94)	(2.32)	(1.75)	(1.21)	(1.17)	(0.46)	(4.05)	(2.06)	(1.87)	(0.47)	(1.16)	(0.88)	(0.60)	(0.58)	(0.23)	(2.03)	(1.03)	(0.93)
C10:0	28.20^*^	38.25^*^	33.18	33.50	33.69	27.19^*^	32.18^*^	32.28^*^	20.02^*^	35.90^*^	30.87^*^	33.41	33.25	33.15	36.41^*^	33.91	33.86	39.99^*^
	(0.95)	(2.05)	(2.13)	(0.59)	(0.92)	(0.71)	(3.55)	(1.96)	(1.67)	(0.47)	(1.02)	(1.07)	(0.30)	(0.46)	(0.36)	(1.77)	(0.98)	(0.83)
C12:0	36.47^*^	57.61^*^	45.46^*^	44.85^*^	41.71^*^	38.55^*^	52.90^*^	45.49^*^	39.25^*^	31.76^*^	21.19^*^	27.27^*^	27.57^*^	29.15^*^	30.72^*^	23.55^*^	27.26^*^	30.38^*^
	(0.86)	(4.21)	(1.82)	(0.83)	(0.80)	(1.11)	(3.97)	(2.36)	(1.25)	(0.43)	(2.10)	(0.91)	(0.42)	(0.40)	(0.56)	(1.98)	(1.18)	(0.62)
C14:0	53.49^*^	78.39^*^	52.02^*^	62.63^*^	59.72^*^	47.71^*^	65.72^*^	56.28^*^	48.48^*^	23.25^*^	10.81^*^	23.99^*^	18.69^*^	20.14^*^	26.14^*^	17.14^*^	21.86^*^	25.76^*^
	(1.21)	(4.52)	(2.36)	(0.96)	(1.59)	(0.61)	(2.50)	(1.17)	(3.05)	(0.61)	(2.26)	(1.18)	(0.48)	(0.80)	(0.31)	(1.25)	(0.59)	(1.53)
C16:0	42.06^*^	44.30^*^	48.56^*^	36.62^*^	33.93	33.44^*^	47.21^*^	44.26^*^	55.48^*^	28.97^*^	27.85^*^	25.72^*^	31.69^*^	33.04^*^	33.28	26.40^*^	27.87^*^	22.26^*^
	(1.25)	(3.91)	(4.56)	(1.58)	(0.49)	(0.92)	(4.80)	(0.34)	(2.92)	(0.62)	(1.95)	(2.28)	(0.79)	(0.25)	(0.46)	(2.40)	(0.17)	(1.46)
C18:0	23.41^*^	26.88^*^	25.36^*^	23.21^*^	30.79^*^	15.41^*^	44.11^*^	41.67^*^	18.30^*^	38.30^*^	36.56^*^	37.32^*^	38.39^*^	34.61^*^	42.29^*^	27.94^*^	29.16^*^	40.85^*^
	(1.43)	(1.83)	(2.29)	(0.95)	(1.50)	(0.08)	(3.42)	(2.11)	(4.36)	(0.72)	(0.92)	(1.14)	(0.47)	(0.75)	(0.04)	(1.71)	(1.05)	(2.18)
C20:0	33.41	17.84^*^	31.78^*^	22.60^*^	46.37^*^	15.21^*^	100.00	46.15^*^	0	33.30	41.08^*^	34.11^*^	38.70^*^	26.82^*^	42.39^*^	0	26.92^*^	50.00
	(3.23)	(3.62)	(4.20)	(1.27)	(3.19)	(1.74)	(0)	(4.37)	(0)	(1.62)	(1.81)	(2.10)	(0.64)	(1.59)	(0.87)	(0)	(2.19)	(0)
C22:0	48.76^*^	28.61^*^	50.37^*^	9.73^*^	75.27^*^	7.30^*^	100.00	0	73.98^*^	25.62^*^	35.70^*^	24.82^*^	45.14^*^	12.37^*^	46.35^*^	0	0	13.01^*^
	(3.56)	(2.26)	(3.61)	(1.89)	(2.37)	(1.11)	(0)	(0)	(2.44)	(1.78)	(1.13)	(1.81)	(0.94)	(1.18)	(0.55)	(0)	(0)	(1.22)
C24:0	45.43^*^	0	51.29^*^	7.50^*^	54.50^*^	9.42^*^	0	0	93.78^*^	27.28^*^	0	24.36^*^	46.25^*^	22.75^*^	45.29^*^	0	0	3.11^*^
	(4.03)	(0)	(2.29)	(0.31)	(1.87)	(1.70)	(0)	(0)	(4.61)	(2.02)	(0)	(1.15)	(0.16)	(0.94)	(0.85)	(0)	(0)	(2.30)
OCSFA																		
C5:0	0	0	0	0	0	0	0	0	0	50.00	50.00	50.00	50.00	50.00	0	0	0	0
	(0)	(0)	(0)	(0)	(0)	(0)	(0)	(0)	(0)	(0)	(0)	(0)	(0)	(0)	(0)	(0)	(0)	(0)
C7:0	6.32^*^	9.12^*^	7.43^*^	11.58^*^	7.76^*^	0	0	17.75^*^	0	46.84^*^	45.44^*^	46.28^*^	44.21^*^	46.12^*^	0	0	41.12^*^	0
	(0.33)	(1.55)	(1.82)	(1.09)	(0.25)	(0)	(0)	(0.63)	(0)	(0.17)	(0.78)	(0.91)	(0.54)	(0.13)	(0)	(0)	(0.31)	(0)
C9:0	24.07^*^	27.22^*^	33.21	34.91^*^	27.76^*^	18.90^*^	36.89^*^	28.95^*^	37.4^*^	37.97^*^	36.39^*^	33.39	32.54^*^	36.12^*^	40.55^*^	31.56^*^	35.53^*^	31.3^*^
	(1.99)	(2.52)	(3.95)	(2.33)	(1.25)	(4.12)	(1.34)	(2.86)	(4.16)	(1.00)	(1.26)	(1.98)	(1.16)	(0.62)	(2.06)	(0.67)	(1.43)	(2.08)
C11:0	32.14^*^	36.81^*^	48.06^*^	68.47^*^	38.71^*^	12.84^*^	58.78^*^	0	40.76^*^	33.93	31.6^*^	25.97^*^	15.77^*^	30.64^*^	43.58^*^	20.61^*^	50.00	29.62^*^
	(0.61)	(2.40)	(2.20)	(4.47)	(2.05)	(2.19)	(3.34)	(0)	(1.16)	(0.31)	(1.20)	(1.10)	(2.23)	(1.03)	(1.10)	(1.67)	(0)	(0.58)
C13:0	52.16^*^	37.93^*^	65.41^*^	54.07^*^	51.08^*^	43.25^*^	0	100.00	50.01^*^	23.92^*^	31.03^*^	17.29^*^	22.97^*^	24.46^*^	28.38^*^	50.00^*^	0	25.00^*^
	(4.49)	(3.27)	(4.10)	(3.56)	(4.94)	(3.47)	(0)	(0)	(1.99)	(2.25)	(1.63)	(2.05)	(1.78)	(2.47)	(1.74)	(0)	(0)	(1.00)
C15:0	41.53^*^	45.36^*^	55.43^*^	50.04^*^	42.25^*^	45.74^*^	79.38^*^	100.00	57.48^*^	29.23^*^	27.32^*^	22.29^*^	24.98^*^	28.87^*^	27.13^*^	10.31^*^	0	21.26^*^
	(0.28)	(0.88)	(1.28)	(0.27)	(1.29)	(0.78)	(1.46)	(0)	(3.92)	(0.14)	(0.44)	(0.64)	(0.13)	(0.65)	(0.39)	(0.73)	(0)	(1.96)
C17:0	37.00^*^	53.35^*^	51.36^*^	46.78^*^	43.80^*^	33.36	100.00	100.00	58.42^*^	31.50^*^	23.32^*^	24.32^*^	26.61^*^	28.10^*^	33.32	0	0	20.79^*^
	(0.66)	(2.94)	(3.20)	(3.65)	(1.39)	(1.14)	(0)	(0)	(2.83)	(0.33)	(1.47)	(1.60)	(1.82)	(0.69)	(0.57)	(0)	(0)	(1.41)
C21:0	100.00	51.98^*^	52.70^*^	7.13^*^	79.95^*^	4.08^*^	0	0	0	0	24.01^*^	23.65^*^	46.44^*^	10.02^*^	47.96^*^	0	0	50.00
	(0)	(4.13)	(4.78)	(1.00)	(2.37)	(0.38)	(0)	(0)	(0)	(0)	(2.06)	(2.39)	(0.50)	(1.18)	(0.19)	(0)	(0)	(0)
C23:0	31.93^*^	11.87^*^	40.29^*^	3.83^*^	59.04^*^	2.83^*^	0	0	0	34.03^*^	44.06^*^	29.85^*^	48.09^*^	20.48^*^	48.59^*^	0	0	50.00
	(2.17)	(1.15)	(4.72)	(0.90)	(3.99)	(1.09)	(0)	(0)	(0)	(1.09)	(0.58)	(2.36)	(0.45)	(1.99)	(0.54)	(0)	(0)	(0)
BCSFA																		
iso C13:0	74.16^*^	68.17^*^	74.64^*^	38.26^*^	51.91^*^	46.02^*^	0	100.00	0	12.92^*^	15.92^*^	12.68^*^	30.87^*^	24.05^*^	26.99^*^	50.00	0	50.00
	(2.57)	(3.16)	(4.13)	(3.94)	(1.05)	(0.67)	(0)	(0)	(0)	(1.29)	(1.58)	(2.06)	(1.97)	(0.52)	(0.33)	(0)	(0)	(0)
anteiso C13:0	61.41^*^	93.63^*^	75.42^*^	44.45^*^	57.43^*^	64.95^*^	0	0	0	19.29^*^	3.18^*^	12.29^*^	27.78^*^	21.28^*^	17.53^*^	0	0	0
	(3.51)	(3.19)	(3.92)	(4.72)	(1.48)	(3.07)	(0)	(0)	(0)	(1.76)	(1.60)	(1.96)	(2.36)	(0.74)	(1.53)	(0)	(0)	(0)
iso C14:0	47.05^*^	77.15^*^	56.86^*^	42.70^*^	59.32^*^	37.55^*^	0	94.13^*^	0	26.47^*^	11.43^*^	21.57^*^	28.65^*^	20.34^*^	31.23^*^	50.00	2.93^*^	50.00
	(1.62)	(3.16)	(4.75)	(1.54)	(1.46)	(2.14)	(0)	(3.10)	(0)	(0.81)	(1.58)	(2.37)	(0.77)	(0.73)	(1.07)	(0)	(1.55)	(0)
iso C15:0	45.99^*^	48.82^*^	49.78^*^	52.18^*^	49.83^*^	34.33^*^	0	0	0	27.01^*^	25.59^*^	25.11^*^	23.91^*^	25.09^*^	32.84^*^	50.00	50.00	50.00
	(1.20)	(2.47)	(2.56)	(3.45)	(4.23)	(1.06)	(0)	(0)	(0)	(0.60)	(1.24)	(1.28)	(1.72)	(2.11)	(0.53)	(0)	(0)	(0)
anteiso C15:0	38.67^*^	44.47^*^	45.08^*^	54.67^*^	45.61^*^	32.77^*^	0	0	58.07^*^	30.66^*^	27.77^*^	27.46^*^	22.66^*^	27.19^*^	33.61^*^	50.00	0	20.96^*^
	(2.40)	(2.39)	(3.30)	(1.87)	(1.63)	(0.96)	(0)	(0)	(3.38)	(1.20)	(1.19)	(1.65)	(0.94)	(0.81)	(0.48)	(0)	(0)	(1.69)
iso C16:0	41.70^*^	43.66^*^	42.71^*^	29.98^*^	41.51^*^	32.08^*^	100.00	97.05^*^	0	29.15^*^	28.17^*^	28.64^*^	35.01^*^	29.24^*^	33.96	0	1.48^*^	50.00
	(2.22)	(2.42)	(3.02)	(1.02)	(3.67)	(0.87)	(0)	(1.65)	(0)	(1.11)	(1.21)	(1.51)	(0.51)	(1.83)	(0.43)	(0)	(0.82)	(0)
iso C17:0	28.25^*^	25.34^*^	32.15^*^	26.17^*^	30.84^*^	21.62^*^	0	100.00	0	35.88^*^	37.33^*^	33.93	36.92^*^	34.58^*^	39.19^*^	0	0	50.00
	(0.49)	(0.37)	(1.59)	(1.82)	(3.49)	(0.62)	(0)	(0)	(0)	(0.25)	(0.18)	(0.80)	(0.91)	(1.75)	(0.31)	(0)	(0)	(0)
anteiso C17:0	32.22^*^	37.34^*^	33.66	27.52^*^	31.27^*^	36.47^*^	100.00	50.87^*^	66.77^*^	33.89	31.33^*^	33.17^*^	36.24^*^	34.36^*^	31.76^*^	0	24.56^*^	16.62^*^
	(0.73)	(2.76)	(1.80)	(1.65)	(0.27)	(0.53)	(0)	(3.48)	(1.04)	(0.37)	(1.38)	(0.90)	(0.83)	(0.13)	(0.27)	(0)	(1.74)	(0.52)
iso C18:0	0	0	0	0	0	0	0	0	0	50.00	50.00	50.00	50.00	50.00	50.00	50.00	50.00	0
	(0)	(0)	(0)	(0)	(0)	(0)	(0)	(0)	(0)	(0)	(0)	(0)	(0)	(0)	(0)	(0)	(0)	0
UFA																		
MUFA																		
C10:1 c9	31.87^*^	64.46^*^	41.29^*^	55.18^*^	42.75^*^	17.63^*^	19.71^*^	40.97^*^	0	34.07^*^	17.77^*^	29.36^*^	22.41^*^	28.62^*^	41.19^*^	40.14^*^	29.52^*^	50.00
	(1.33)	(3.16)	(0.92)	(2.31)	(0.71)	(1.00)	(2.96)	(3.07)	(0)	(0.67)	(1.58)	(0.46)	(1.16)	(0.36)	(0.50)	(1.48)	(1.53)	(0)
C14:1 c9	36.36^*^	59.37^*^	53.69^*^	49.09^*^	45.97^*^	44.06^*^	0	100.00	0	31.82^*^	20.31^*^	23.16^*^	25.46^*^	27.02^*^	27.97^*^	50.00	0	50.00
	(1.87)	(4.25)	(4.44)	(1.94)	(4.67)	(1.63)	(0)	(0)	(0)	(0.94)	(2.13)	(2.22)	(0.97)	(2.34)	(0.81)	(0)	(0)	(0)
C16:1 t9	0	33.24	44.87^*^	20.45^*^	0	0	0	0	0	0	33.38	27.56^*^	39.78^*^	50.00	50.00	0	0	0
	(0)	(3.96)	(4.33)	(0.86)	(0)	(0)	(0)	(0)	(0)	(0)	(1.98)	(2.16)	(0.43)	(0)	(0)	(0)	(0)	(0)
C16:1 c7	21.06^*^	17.52^*^	25.03^*^	17.31^*^	26.33^*^	18.56^*^	41.09^*^	39.79^*^	36.85^*^	39.47^*^	41.24^*^	37.48^*^	41.34^*^	36.84^*^	40.72^*^	29.46^*^	30.10^*^	31.57^*^
	(1.10)	(1.87)	(1.34)	(1.79)	(4.57)	(1.15)	(1.02)	(2.12)	(3.97)	(0.55)	(0.94)	(0.67)	(0.89)	(2.29)	(0.58)	(0.51)	(1.06)	(1.99)
C16:1 c9	30.30^*^	42.57^*^	37.10^*^	32.85^*^	38.54^*^	42.22^*^	35.64^*^	28.98^*^	32.90^*^	34.85^*^	28.71^*^	31.45^*^	33.58	30.73^*^	28.89^*^	32.18^*^	35.51^*^	33.55
	(0.87)	(4.37)	(0.99)	(0.84)	(2.83)	(2.03)	(0.11)	(1.24)	(3.89)	(0.43)	(2.19)	(0.49)	(0.42)	(1.42)	(1.02)	(0.06)	(0.62)	(1.95)
C17:1 c10	0	0	0	0	0	0	0	0	0	0	50.00	0	50.00	0	50.00	0	0	0
	(0)	(0)	(0)	(0)	(0)	(0)	(0)	(0)	(0)	(0)	(0)	(0)	(0)	(0)	(0)	(0)	(0)	(0)
C18:1 t6	34.19^*^	20.99^*^	39.84^*^	12.66^*^	33.3	37.31^*^	0	0	0	32.90	39.50^*^	30.08^*^	43.67^*^	33.35	31.34^*^	50.00	0	50.00
	(3.19)	(3.11)	(3.41)	(1.32)	(3.82)	(2.20)	(0)	(0)	(0)	(1.59)	(1.56)	(1.70)	(0.66)	(1.91)	(1.10)	(0)	(0)	(0)
C18:1 t9	100.00	0	27.03^*^	14.45^*^	30.95^*^	0	0	0	0	0	50.00	36.48^*^	42.78^*^	34.52^*^	50.00	50.00	0	50.00
	(0)	(0)	(2.22)	(1.28)	(4.06)	(0)	(0)	(0)	(0)	(0)	(0)	(1.11)	(0.64)	(2.03)	(0)	(0)	(0)	(0)
C18:1 t11	21.60^*^	26.30^*^	24.01^*^	24.39^*^	30.51^*^	15.02^*^	0	0	25.28^*^	39.20^*^	36.85^*^	38.00^*^	37.81^*^	34.75^*^	42.49^*^	50.00	0	37.36^*^
	(0.87)	(3.03)	(2.87)	(0.97)	(4.38)	(2.05)	(0)	(0)	(3.61)	(0.43)	(1.51)	(1.43)	(0.48)	(2.19)	(1.03)	(0)	(0)	(1.80)
C18:1 c6	12.00^*^	16.89^*^	34.41^*^	12.56^*^	28.24^*^	7.12^*^	0	0	0	44.00^*^	41.55^*^	32.79^*^	43.72^*^	35.88^*^	46.44^*^	50.00	0	50.00
	(1.08)	(2.86)	(4.59)	(0.28)	(4.27)	(0.50)	(0)	(0)	(0)	(0.54)	(1.43)	(2.29)	(0.14)	(2.13)	(0.25)	(0)	(0)	(0)
C18:1 c9	34.84^*^	45.21^*^	47.27^*^	34.32^*^	45.78^*^	41.47^*^	23.15^*^	21.07^*^	28.28^*^	32.58^*^	27.40^*^	26.37^*^	32.84^*^	27.11^*^	29.26^*^	38.43^*^	39.46^*^	35.86^*^
	(1.19)	(1.90)	(1.00)	(3.26)	(2.90)	(1.37)	(0.45)	(0.87)	(2.27)	(0.60)	(0.95)	(0.50)	(1.63)	(1.45)	(0.68)	(0.23)	(0.44)	(1.13)
C18:1 c11	12.08^*^	4.18^*^	26.58^*^	6.27^*^	17.16^*^	8.31^*^	27.13^*^	13.83^*^	21.77^*^	43.96^*^	47.91^*^	36.71^*^	46.87^*^	41.42^*^	45.84^*^	36.43^*^	43.08^*^	39.11^*^
	(0.57)	(0.25)	(4.73)	(1.9)	(4.00)	(0.32)	(3.25)	(0.54)	(2.68)	(0.29)	(0.12)	(2.37)	(0.95)	(2.00)	(0.16)	(1.63)	(0.27)	(1.34)
C18:1 c12	20.08^*^	30.10^*^	34.53^*^	32.78^*^	32.06^*^	18.01^*^	0	0	0	39.96^*^	34.95^*^	32.73^*^	33.61^*^	33.97^*^	40.99^*^	50.00	0	50.00
	(2.59)	(3.35)	(3.34)	(1.28)	(4.22)	(0.98)	(0)	(0)	(0)	(1.29)	(1.68)	(1.67)	(0.64)	(2.11)	(0.49)	(0)	(0)	(0)
C20:1 t11	0	0	0	0	0	0	0	0	0	0	0	0	0	50	0	0	0	0
	(0)	(0)	(0)	(0)	(0)	(0)	(0)	(0)	(0)	(0)	(0)	(0)	(0)	(0)	(0)	(0)	(0)	(0)
C20:1 c8	58.87^*^	44.70^*^	51.25^*^	0	0	0	0	0	0	20.57^*^	27.65^*^	24.38^*^	0	0	50.00	0	0	0
	(5.24)	(2.51)	(3.34)	(0)	(0)	(0)	(0)	(0)	(0)	(2.62)	(1.26)	(1.67)	(0)	(0)	(0)	(0)	(0)	(0)
C20:1 c11	0	0	0	13.6^*^	55.88^*^	28.95^*^	48.24^*^	24.46^*^	52.34^*^	0	0	0	43.20^*^	22.06^*^	35.53^*^	25.88^*^	37.77^*^	23.83^*^
	(0)	(0)	(0)	(0.70)	(2.35)	(2.95)	(1.51)	(4.35)	(2.96)	(0)	(0)	(0)	(0.35)	(1.18)	(1.48)	(0.76)	(2.17)	(1.48)
C22:1 t13	0	0	0	0	0	0	0	0	0	0	0	0	0	0	0	0	0	0
	(0)	(0)	(0)	(0)	(0)	(0)	(0)	(0)	(0)	(0)	(0)	(0)	(0)	(0)	(0)	(0)	(0)	(0)
C22:1 c13	0	0	0	0	0	0	0	0	0	0	0	0	50.00	0	50.00	0	0	0
	(0)	(0)	(0)	(0)	(0)	(0)	(0)	(0)	(0)	(0)	(0)	(0)	(0)	(0)	(0)	(0)	(0)	(0)
C24:1 c15	0	0	0	0	0	0	0	0	0	0	0	50.00	0	50.00	0	50.00	0	50.00
	(0)	(0)	(0)	(0)	(0)	(0)	(0)	(0)	(0)	(0)	(0)	(0)	(0)	(0)	(0)	(0)	(0)	(0)
PUFA																		
C18:2 t9t12	0	0	0	0	0	0	0	0	0	0	0	0	50.00	0	50.00	0	0	0
	(0)	(0)	(0)	(0)	(0)	(0)	(0)	(0)	(0)	(0)	(0)	(0)	(0)	(0)	(0)	(0)	(0)	(0)
C18:2 c9t12	0	0	0	0	0	0	0	0	0	50.00	50.00	50.00	50.00	50.00	50.00	50.00	50.00	50.00
	(0)	(0)	(0)	(0)	(0)	(0)	(0)	(0)	(0)	(0)	(0)	(0)	(0)	(0)	(0)	(0)	(0)	(0)
C18:2 t9c12	0	0	0	0	0	11.93^*^	0	0	0	0	50.00	0	0	50.00	44.03^*^	0	0	50.00
	(0)	(0)	(0)	(0)	(0)	(2.73)	(0)	(0)	(0)	(0)	(0)	(0)	(0)	(0)	(1.37)	(0)	(0)	(0)
C18:2 c9c12	36.46^*^	40.40^*^	37.76^*^	40.73^*^	43.91^*^	53.53^*^	27.49^*^	33.67^*^	23.31^*^	31.77^*^	29.8^*^	31.12^*^	29.64^*^	28.04^*^	23.24^*^	36.25^*^	33.16	38.34^*^
	(1.06)	(3.62)	(3.29)	(1.57)	(1.61)	(1.98)	(2.45)	(1.45)	(4.40)	(0.53)	(1.81)	(1.64)	(0.79)	(0.81)	(0.99)	(1.22)	(0.73)	(2.20)
C18:3 c6c9c12	31.07^*^	10.40^*^	44.92^*^	4.10^*^	41.31^*^	2.09^*^	0	0	20.13^*^	34.47^*^	44.80^*^	27.54^*^	47.95^*^	29.34^*^	48.96^*^	50.00	50.00	39.94^*^
	(1.54)	(2.12)	(3.93)	(1.50)	(4.66)	(0.54)	(0)	(0)	(4.82)	(0.77)	(1.06)	(1.96)	(0.75)	(2.33)	(0.27)	(0)	(0)	(2.41)
C18:3 c9c12c15	33.07^*^	24.69^*^	38.24^*^	18.04^*^	31.86^*^	39.68^*^	49.45^*^	18.48^*^	12.65^*^	33.47	37.66^*^	30.88^*^	40.98^*^	34.07^*^	30.16^*^	25.28^*^	40.76^*^	43.68^*^
	(4.50)	(4.87)	(4.55)	(4.48)	(1.50)	(1.33)	(2.72)	(0.75)	(2.15)	(2.25)	(2.44)	(2.28)	(2.24)	(0.75)	(0.67)	(1.36)	(0.38)	(1.08)
C18:2 c9t11	30.28^*^	38.76^*^	31.25^*^	48.26^*^	42.54^*^	41.95^*^	0	0	0	34.86^*^	30.62^*^	34.37^*^	25.87^*^	28.73^*^	29.02^*^	50.00	50.00	50.00
	(3.80)	(2.81)	(4.52)	(4.10)	(3.38)	(3.09)	(0)	(0)	(0)	(1.90)	(1.41)	(2.26)	(2.05)	(1.69)	(1.54)	(0)	(0)	(0)
C18:2 c9c11	0	15.86^*^	100.00	0	0	14.83^*^	0	0	0	0	42.07^*^	0	0	0	42.59^*^	0	0	0
	(0)	(3.82)	(0)	(0)	(0)	(4.22)	(0)	(0)	(0)	(0)	(1.91)	(0)	(0)	(0)	(2.11)	(0)	(0)	(0)
C20:2 c11c14	58.97^*^	0	96.79^*^	0	50.80^*^	6.27^*^	60.31^*^	0	57.73^*^	20.51^*^	0	1.61^*^	0	24.60^*^	46.87^*^	19.85^*^	0	21.14^*^
	(1.41)	(0)	(1.96)	(0)	(4.83)	(1.03)	(2.24)	(0)	(3.03)	(0.70)	(0)	(0.98)	(0)	(2.42)	(0.52)	(1.12)	(0)	(1.52)
C20:3 c8c11c14	47.55^*^	22.32^*^	62.21^*^	2.63^*^	64.38^*^	6.17^*^	0	24.09^*^	71.21^*^	26.22^*^	38.84^*^	18.89^*^	48.68^*^	17.81^*^	46.92^*^	50.00	37.96^*^	14.40^*^
	(1.20)	(3.11)	(1.42)	(0.84)	(1.28)	(0.73)	(0)	(3.76)	(3.54)	(0.60)	(1.55)	(0.71)	(0.42)	(0.64)	(0.36)	(0)	(1.88)	(1.77)
C20:3 c11c14c17	0	0	19.68^*^	0	0	0	0	20.44^*^	0	0	50.00	40.16^*^	0	0	50.00	50.00	39.78^*^	50.00
	(0)	(0)	(4.35)	(0)	(0)	(0)	(0)	(2.23)	(0)	(0)	(0)	(2.17)	(0)	(0)	(0)	(0)	(1.11)	(0)
C20:4 c5c8c11c14	30.30^*^	23.23^*^	31.78^*^	6.62^*^	38.45^*^	10.40^*^	0	30.54^*^	37.22^*^	34.85^*^	38.39^*^	34.11^*^	46.69^*^	30.78^*^	44.80^*^	50.00	34.73^*^	31.39^*^
	(1.62)	(2.92)	(2.45)	(0.82)	(4.64)	(1.21)	(0)	(1.61)	(1.86)	(0.81)	(1.46)	(1.23)	(0.41)	(2.32)	(0.61)	(0)	(0.80)	(0.93)
C22:2 c13c16	0	0	0	0	0	0	0	0	0	0	0	0	0	0	0	0	0	50.00
	(0)	(0)	(0)	(0)	(0)	(0)	(0)	(0)	(0)	(0)	(0)	(0)	(0)	(0)	(0)	(0)	(0)	(0)
C20:5 c5c8c11c14c17	62.66^*^	0	45.03^*^	5.09^*^	0	23.13^*^	0	0	68.23^*^	18.67^*^	0	27.49^*^	47.46^*^	50.00	38.43^*^	0	0	15.88^*^
	(0.92)	(0)	(4.93)	(1.18)	(0)	(3.53)	(0)	(0)	(2.45)	(0.46)	(0)	(2.47)	(0.59)	(0)	(1.76)	(0)	(0)	(1.23)
C22:3 c13c16c19	0	0	0	0	0	0	0	0	0	0	0	0	0	0	0	0	50	0
	(0)	(0)	(0)	(0)	(0)	(0)	(0)	(0)	(0)	(0)	(0)	(0)	(0)	(0)	(0)	(0)	(0)	(0)
C22:4 c7c10c13c16	77.59^*^	0	85.70^*^	5.56^*^	80.80^*^	12.75^*^	0	0	92.07^*^	11.21^*^	0	7.15^*^	47.22^*^	9.60^*^	43.63^*^	0	0	3.97^*^
	(5.47)	0	(4.62)	(1.24)	(4.43)	(2.15)	0	0	(3.97)	(2.74)	0	(2.31)	(0.62)	(2.22)	(1.07)	0	0	(1.98)
C22:5 c4c7c10c13c16	0	0	100.00	3.33^*^	73.77^*^	100.00	0	0	81.20^*^	50.00	0	0	48.33^*^	13.11^*^	0	0	0	9.40^*^
	(0)	(0)	(0)	(1.20)	(3.31)	(0)	(0)	(0)	(4.88)	(0)	(0)	(0)	(0.60)	(1.66)	(0)	(0)	(0)	(2.44)
C22:5 c7c10c13c16c19	93.37^*^	70.46^*^	57.58^*^	12.54^*^	94.83^*^	44.28^*^	0	0	63.59^*^	3.31^*^	14.77^*^	21.21^*^	43.73^*^	2.59^*^	27.86^*^	0	0	18.21^*^
	(2.08)	(2.61)	(4.82)	(2.61)	(2.30)	(1.49)	(0)	(0)	(1.96)	(1.04)	(1.31)	(2.41)	(1.31)	(1.15)	(0.74)	(0)	(0)	(0.98)
C22:6 c4c7c10c13c16c19	0	0	70.92^*^	2.67^*^	93.98^*^	0	0	100.00	61.79^*^	0	0	14.54^*^	48.66^*^	3.01^*^	0	0	0	19.10^*^
	(0)	(0)	(3.91)	(0.70)	(2.48)	(0)	(0)	(0)	(3.85)	(0)	(0)	(1.95)	(0.35)	(1.24)	(0)	(0)	(0)	(1.92)
FA group																		
SFA	33.20	31.19^*^	30.02^*^	34.56^*^	29.98^*^	31.90^*^	44.07^*^	43.03^*^	45.21^*^	33.40	34.41^*^	34.99^*^	32.72^*^	35.01^*^	34.05^*^	27.97^*^	28.48^*^	27.39^*^
	(0.35)	(0.71)	(0.70)	(0.68)	(0.68)	(0.61)	(2.06)	(1.12)	(2.55)	(0.17)	(0.36)	(0.35)	(0.34)	(0.34)	(0.30)	(1.03)	(0.56)	(1.27)
ECSFA	33.06	30.83^*^	29.17^*^	34.46^*^	29.55^*^	31.67^*^	43.60^*^	42.54^*^	45.15^*^	33.47	34.59^*^	35.42^*^	32.77^*^	35.23^*^	34.16^*^	28.20^*^	28.73^*^	27.43^*^
	(0.35)	(0.75)	(0.74)	(0.65)	(0.66)	(0.62)	(2.12)	(1.12)	(2.55)	(0.18)	(0.38)	(0.37)	(0.33)	(0.33)	(0.31)	(1.06)	(0.56)	(1.28)
OCSFA	37.17^*^	41.28^*^	52.14^*^	39.73^*^	40.85^*^	38.58^*^	76.89^*^	90.52^*^	55.49^*^	31.42^*^	29.36^*^	23.93^*^	30.14^*^	29.57^*^	30.71^*^	11.56^*^	4.74^*^	22.26^*^
	(0.96)	(1.26)	(1.86)	(2.25)	(1.20)	(0.71)	(1.23)	(1.29)	(2.54)	(0.48)	(0.63)	(0.93)	(1.12)	(0.60)	(0.36)	(0.62)	(0.65)	(1.27)
BCSFA	36.99^*^	43.61^*^	40.43^*^	34.56^*^	39.38^*^	32.39	73.56^*^	69.00^*^	35.88^*^	31.50^*^	28.19^*^	29.78^*^	32.72^*^	30.31^*^	33.81	13.22^*^	15.50^*^	32.06^*^
	(1.05)	(1.96)	(2.64)	(1.17)	(2.03)	(0.68)	(3.64)	(1.90)	(1.15)	(0.52)	(0.98)	(1.32)	(0.59)	(1.02)	(0.34)	(1.82)	(0.95)	(0.58)
UFA	33.62	39.12^*^	43.40^*^	29.80^*^	43.57^*^	35.98^*^	26.01^*^	23.32^*^	26.17^*^	33.19^*^	30.44^*^	28.30^*^	35.10^*^	28.21^*^	32.01^*^	36.99^*^	38.34^*^	36.92^*^
	(0.88)	(1.87)	(0.77)	(2.25)	(1.62)	(1.10)	(1.05)	(0.69)	(1.07)	(0.44)	(0.94)	(0.39)	(1.13)	(0.81)	(0.55)	(0.53)	(0.35)	(0.54)
n-1 UFA	31.87^*^	64.46^*^	41.29^*^	55.18^*^	42.75^*^	17.63^*^	19.71^*^	40.97^*^	0	34.07^*^	17.77^*^	29.36^*^	22.41^*^	28.62^*^	41.19^*^	40.14^*^	29.52^*^	50.00
	(1.33)	(3.16)	(0.92)	(2.31)	(0.71)	(1.00)	(2.96)	(3.07)	(0)	(0.67)	(1.58)	(0.46)	(1.16)	(0.36)	(0.50)	(1.48)	(1.53)	(0)
n-3 UFA	36.25*	22.49*	38.77*	12.45*	36.63*	36.32*	47.06*	18.51*	15.60*	31.88*	38.76*	30.61*	43.78*	31.68*	31.84*	26.47*	40.75*	42.20*
	(4.30)	(4.20)	(4.36)	(2.29)	(3.00)	(1.84)	(2.10)	(0.73)	(2.37)	(2.15)	(2.10)	(2.18)	(1.14)	(1.50)	(0.92)	(1.05)	(0.37)	(1.18)
n-5 UFA	36.36*	59.37*	53.69*	49.09*	45.97*	44.06*	0	100.00	0	31.82*	20.31*	23.16*	25.46*	27.02*	27.97*	50.00	0	50.00
	(1.87)	(4.25)	(4.44)	(1.94)	(4.67)	(1.63)	(0)	(0)	(0)	(0.94)	(2.13)	(2.22)	(0.97)	(2.34)	(0.81)	(0)	(0)	(0)
n-6 UFA	36.90*	29.88*	38.53*	19.93*	42.71*	26.85*	27.51*	31.63*	25.26*	31.55*	35.06*	30.74*	40.04*	28.65*	36.57*	36.24*	34.18*	37.37*
	(1.10)	(2.72)	(3.16)	(3.35)	(1.67)	(3.36)	(2.44)	(0.62)	(4.31)	(0.55)	(1.36)	(1.58)	(1.68)	(0.84)	(1.68)	(1.22)	(0.31)	(2.15)
n-7 UFA	24.17*	27.05*	30.00	21.51*	31.17*	30.15*	33.17	27.05*	26.52*	37.92*	36.47*	35.00*	39.25*	34.41*	34.92*	33.42	36.48*	36.74*
	(0.46)	(1.29)	(2.59)	(0.49)	(4.66)	(0.77)	(0.91)	(0.90)	(2.47)	(0.23)	(0.65)	(1.30)	(0.25)	(2.33)	(0.38)	(0.45)	(0.45)	(1.23)
n-9 UFA	34.83*	44.53*	46.85*	33.45	45.43*	40.23*	23.60*	21.36*	28.45*	32.59*	27.74*	26.58*	33.27	27.28*	29.89*	38.20*	39.32*	35.78*
	(1.18)	(1.89)	(1.01)	(3.09)	(2.76)	(1.25)	(0.41)	(0.94)	(2.26)	(0.59)	(0.95)	(0.50)	(1.55)	(1.38)	(0.62)	(0.20)	(0.47)	(1.13)
n-12 UFA	25.67*	22.30*	42.85*	12.62*	30.99*	23.52*	0	0	0	37.16*	38.85*	28.58*	43.69*	34.51*	38.24*	50.00	0	50.00
	(1.38)	(2.89)	(3.55)	(0.69)	(3.75)	(0.89)	(0)	(0)	(0)	(0.69)	(1.44)	(1.77)	(0.34)	(1.87)	(0.45)	(0)	(0)	(0)
MUFA	33.13	40.22*	44.06*	31.91*	43.64*	36.91*	24.26*	24.45*	28.11*	33.43	29.89*	27.97*	34.04*	28.18*	31.54*	37.87*	37.78*	35.94*
	(0.92)	(1.77)	(0.66)	(2.48)	(1.87)	(0.94)	(0.22)	(0.80)	(2.25)	(0.46)	(0.89)	(0.33)	(1.24)	(0.93)	(0.47)	(0.11)	(0.40)	(1.12)
PUFA	37.49*	30.61*	38.32*	20.42*	42.91*	29.53*	28.15*	21.91*	24.70*	31.26*	34.70*	30.84*	39.79*	28.55*	35.23*	35.93*	39.04*	37.65*
	(1.45)	(2.05)	(4.03)	(3.31)	(1.92)	(2.93)	(2.43)	(0.55)	(4.19)	(0.73)	(1.02)	(2.02)	(1.65)	(0.96)	(1.47)	(1.22)	(0.27)	(2.10)
SCFA	4.25*	2.49*	3.40*	4.27*	3.42*	2.38*	9.80*	10.81*	9.60*	47.88*	48.76*	48.30*	47.87*	48.29*	48.81*	45.10*	44.60*	45.20*
	(0.70)	(0.07)	(0.31)	(0.40)	(0.53)	(0.27)	(2.82)	(1.42)	(2.84)	(0.35)	(0.03)	(0.15)	(0.20)	(0.26)	(0.14)	(1.41)	(0.71)	(1.42)
MCFA	42.88*	66.10*	44.95*	45.71*	46.06*	46.35*	40.76*	42.10*	39.09*	28.56*	16.95*	27.53*	27.14*	26.97*	26.83*	29.62*	28.95*	30.45*
	(0.44)	(2.15)	(1.38)	(0.78)	(1.59)	(0.59)	(1.50)	(1.99)	(1.28)	(0.22)	(1.07)	(0.69)	(0.39)	(0.79)	(0.29)	(0.75)	(0.99)	(0.64)
LCFA	36.45*	39.04*	42.20*	32.46*	37.74*	30.85*	31.80*	30.61*	32.77*	31.78*	30.48*	28.90*	33.77	31.13*	34.57*	34.10*	34.70*	33.62
	(0.72)	(0.52)	(2.35)	(0.74)	(0.88)	(0.11)	(0.45)	(0.54)	(0.14)	(0.36)	(0.26)	(1.18)	(0.37)	(0.44)	(0.05)	(0.22)	(0.27)	(0.07)

^*^Data represent the test of FA esterification preference against the null hypothesis of equal distribution (33.3%). Statistical significance (*P* < 0.05) is indicated. Values without parentheses are means, and values in parentheses are standard errors.

A parallel trend was observed for OCSFA. Human (0.36 mol%), mare (0.29 mol%), and donkey milk (0.35 mol%) contained minimal amounts at the TG level, with correspondingly low proportions at both the sn-2 and sn-1/3 positions. Notably, these non-ruminant milks exhibited an sn-2 preference for OCSFA. This finding partially aligns with the literature, which reports that OCSFA and BCSFA in cow, goat, sheep, and camel milk also tend to esterify at the sn-2 position, whereas in mare milk, they are preferentially located at the sn-1/3 positions^23,31,40^. In the present study, with the exception of camel milk, BCSFA showed a universal sn-2 preference across all species. Donkey and mare milk demonstrated the highest positional specificity (69.00–73.56% at sn-2), despite their low total BCSFA content at the TG level (0.13–0.31 mol%).

The distribution of UFAs revealed further complexity. At the TG level, MUFA were most abundant in camel (30.11 mol%), human (31.89 mol%), and donkey milk (32.57 mol%). Positionally, MUFA in human, donkey, mare, and goat milk preferentially occupied the sn-1/3 positions, while in cow, yak, buffalo, sheep, and camel milk, they were predominantly esterified at the sn-2 position. This observation is consistent with literature reports indicating a general sn-1/3 preference for MUFA across most of these species^23,26,31,37–43^. PUFAs were exceptionally rich in human (29.96 mol%), donkey (26.90 mol%), and mare milk (22.63 mol%) at the TG level, where they showed a strong sn-1/3 positional preference. This contrasted with cow, sheep, and yak milk, where the much lower PUFA levels exhibited an sn-2 preference. It is worth noting that the positional distribution of PUFA in camel and mare milk remains somewhat ambiguous in the literature, with reports suggesting both sn-1/3 and sn-2 preferences^23,26,31,37–40^ .

SCFAs were overwhelmingly abundant in ruminant milks at the TG level (10.34–23.08 mol%) and exhibited a universal sn-1/3 preference across all species. Conversely, MCFA demonstrated a consistent sn-2 preference in all milks, with the highest total content at the TG level found in goat milk (28.27 mol%) and mare milk (25.89 mol%). LCFA, most abundant in human (90.48 mol%) and camel milk (82.17 mol%) at the TG level, were preferentially located at the sn-1/3 positions in human, donkey, mare, goat, and camel milk, but surprisingly at the sn-2 position in cow, buffalo, sheep, and yak milk.

Functionally, these distinct regiospecific profiles have profound implications for neonatal nutrition. The high sn-2 palmitate configuration shared by human, donkey, and mare milk enhances calcium soap formation and improves fat absorption in the infant gut^44^. The sn-1/3 localization of SCFA in ruminant milks facilitates rapid lipolysis and quick energy release, aligning with the growth demands of their precocial offspring. The strategic positioning of PUFA at the sn-1/3 positions in human and donkey milk may optimize the bioavailability of these essential fatty acids for neurological development.

The underlying causes for these structural disparities are deeply rooted in evolutionary biology and enzymology. The convergent sn-2 ECSFA pattern in human, mare, and donkey milk reflects shared biosynthetic constraints in monogastric mammals, governed by the substrate specificity of their acyl-CoA:lysophosphatidic acid acyltransferases (LPAATs)^45^. The contrasting ruminant pattern is a direct metabolic consequence of rumen biohydrogenation, which generates a unique FA profile subsequently processed by phylogenetically related LPAAT isoforms. The distinctive camel milk profile underscores a separate evolutionary trajectory, potentially representing an adaptive optimization for neonatal development in arid environments.

The significantly higher abundance of SFA, particularly SCFA and OCSFA, in ruminant milks compared to human milk is primarily attributable to the distinct anatomical and metabolic features of the ruminant digestive system. In the rumen, extensive microbial fermentation of dietary carbohydrates and fiber yields substantial quantities of volatile FAs, including acetate, propionate, and butyrate^35^. Acetate and butyrate serve as the principal substrates for de novo FA synthesis in the ruminant mammary gland, directly contributing to the elevated levels of short- and medium-chain SFA in milk^46^. In contrast, human mammary tissue relies predominantly on glucose and circulating lipids for milk fat synthesis, resulting in a notably lower SCFA content. Furthermore, the biosynthesis of OCSFA is intimately linked to rumen microbial metabolism. Propionate, a major rumen fermentation product, can be utilized as a primer unit for OCSFA elongation via the FA synthase complex, yielding substantial amounts of C15:0 and C17:0 in milk fat^47^. Additionally, the rumen microbiome produces significant quantities of OCSFA and BCSFA de novo from branched-chain amino acids and other fermentation intermediates, which are subsequently absorbed and incorporated into milk lipids^48^. The low levels of these FAs in human milk reflect the absence of a rumen and a fundamentally different precursor pool derived from the systemic circulation and endogenous synthesis. Therefore, the disparity in SFA, SCFA, and OCSFA concentrations between ruminant and human milks is a direct metabolic signature of the ruminant’s foregut fermentation strategy and its integration with mammary lipid synthesis.

### Lipid similarity evaluation of eight mammalian milk and human milk

**Fig. 5:**
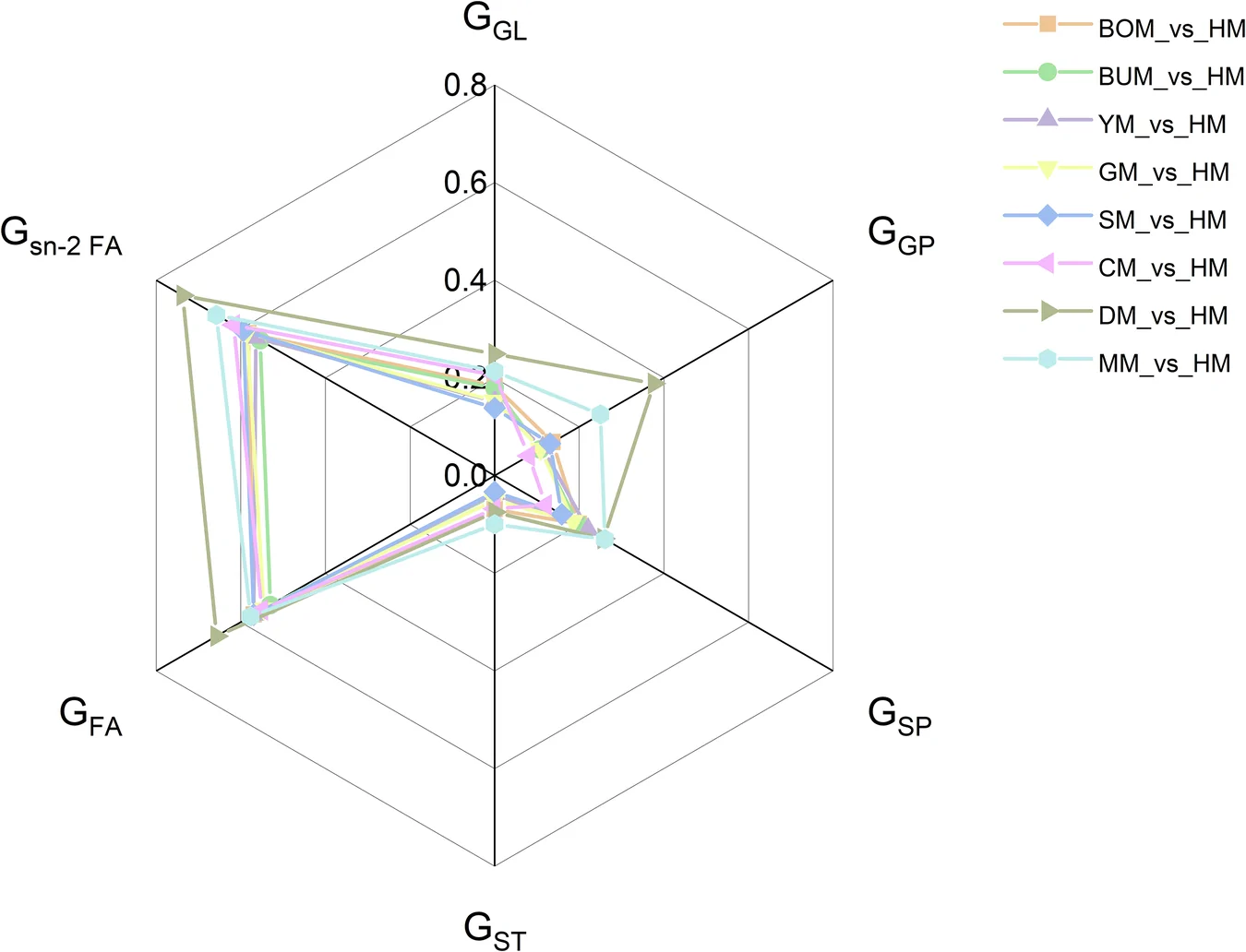
Radar chart of lipid similarity between mammalian milk and human milk.

Human milk lipids represent the gold standard for evaluating potential substitutes. We assessed the similarity of lipids from various animal sources using a multi-criteria model, with Bray–Curtis similarity metric categorized into six groups (Fig. 5). Based on lipid species profile, the similarity scores of the alternative milk sources ranged from 0.13 to 0.25. Donkey milk showed the highest score (0.25), followed by mare milk (0.22). Camel milk and cow milk both scored 0.17, followed by buffalo milk (0.16), yak milk (0.14), goat milk (0.14), and sheep milk (0.13). When the FA composition of TG was used as the criterion, donkey milk again yielded the highest similarity (0.66). The scores for mare, cow, sheep, and yak milk were comparable (0.57-0.58), while camel, goat, and buffalo milk scored lower (0.53-0.55). Assessment of the sn-2 FA profile confirmed donkey milk as the closest match (0.73), followed by mare milk (0.65). The ruminant milks scored between 0.55 and 0.61. The comprehensive similarity score, which integrated all criteria, ranked donkey milk highest (0.55), followed by mare milk (0.48). The ruminant milks scored the lowest (0.41–0.44). The lower similarity scores of ruminant milks compared to monogastric milks (donkey, mare) may be attributed to differences in lipid metabolism. The biosynthetic pathways in monogastric animals more closely resemble those in humans, resulting in a lipid profile more like human milk’ lipid. Currently, infant formula manufacturing often prioritizes total lipid content over specific molecular composition. This focus leads to inconsistent lipid supplementation across products. Our findings highlight the need to strategically identify and combine lipid resources that better mimic the molecular species found in human milk’ lipid. Such an approach would more effectively meet infants’ nutritional requirements for both lipid levels and specific molecular species.

The nutritional value of milk fat is governed not only by its FA composition but also by the regiospecific distribution of FA within TG. This study systematically elucidated the lipid molecular composition and TG regiospecific FA distribution in milk from nine mammalian species: cow, buffalo, yak, goat, sheep, camel, mare, donkey, and human. Comprehensive lipidomic profiling using liquid chromatography–tandem mass spectrometry revealed a relatively high similarity in lipid composition among ruminant milks, which were distinctly different from camel and monogastric milks. Compared to human milk, the eight animal milks exhibited higher relative abundances of TG, PC, PE, and SM, but lower levels of FFAs and DGs. With respect to positional distribution, SCFAs preferentially esterified at the sn-1/3 positions across all species, whereas MCFAs, LCFAs, OCSFAs tended to occupy the sn-2 position. In monogastric milks, palmitic acid and other SFAs were predominantly located at the sn-2 position, while oleic acid, linoleic acid, and polyunsaturated FAs favored the sn-1/3 positions. In contrast, UFAs in ruminant (except goat) and camel milks showed a preference for the sn-2 position, whereas stearic acid was preferentially esterified at the sn-1/3 positions. Bray-Curtis similarity analysis based on both lipid molecular species and sn-2 positional FA profiles consistently identified donkey milk as the closest lipid analog to human milk, followed by mare milk. Collectively, this work provides a systematic characterization of the global lipidome and TG positional architecture of nine mammalian milks. These findings establish a robust scientific basis for the nutritional evaluation of animal milks and offer essential compositional data to guide the humanized lipid design of next-generation infant formulas.

## Methods

### Milk samples processing

Milk samples from nine mammalian species were analyzed: Holstein cow milk (BOM), buffalo milk (BUM), yak milk (YM), goat milk (GM), sheep milk (SM), camel milk (CM), mare milk (MM), donkey milk (DM), and human milk (HM). The collection was non‑invasive, with no dietary or lifestyle intervention for animals or human donors. For non‑human species, samples were obtained as pooled bulk milk from commercial tanker trucks or farm bulk tanks. As colostrum is routinely excluded from such commercial collections, these pooled samples inherently represent mature milk. Each biological replicate (*n* = 3) consisted of pooled milk representing multiple animals rather than individual samples. Specifically, BOM, BUM, GM, and CM samples were sourced from tanker trucks in Shanghai, Guangxi, Shaanxi, and Xinjiang, China, respectively, with each pooled replicate representing approximately 1000–5000 cows, 250–1000 buffaloes, 500–1000 goats, and 50–100 Bactrian camels. YM, SM, MM, and DM samples were collected from bulk milk tanks at cooperatives in Gansu, Inner Mongolia, and Xinjiang, China, with each pooled replicate representing approximately 10–20 lactating animals of the respective species. HM samples were obtained from 18 healthy lactating mothers in Beijing, China. All donors delivered full‑term infants, had no history of metabolic disease, and were 3–6 months postpartum. Written informed consent was obtained from all participants. The 18 individual HM samples were randomly pooled into three biological replicates for analysis. This study was approved by the Animal Ethics Committee of the Institute of Animal Science, Chinese Academy of Agricultural Sciences (Beijing, China; approval no. IAS2022‑124). All milk samples were immediately frozen and stored at −80 °C until analysis.

### Chemicals and reagents

Hexane and isopropanol were purchased from Merck (Darmstadt, Germany). Methanol, dichloromethane, and acetonitrile were obtained from Fisher Scientific (Loughborough, UK). Formic acid was purchased from Sigma-Aldrich (St. Louis, MO, USA). Ammonium formate, diethyl ether, sodium hydroxide, and sodium chloride were obtained from Shanghai Chemical Reagent Co., Ltd (Shanghai, China). Acetyl chloride was obtained from CNW Technologies (Shanghai, China). Ultrapure water was purified using a Milli-Q water system (Millipore, Bedford, MA, USA). Solid-phase extraction cartridges (NH₂ and Si) were purchased from Phenomenex (Torrance, CA, USA). *CALB* (Novozym 435, 10,000 propyl laurate units/g) was obtained from Novozymes A/S (Bagsværd, Denmark). A standard of TG C19:0_C19:0_C19:0 was purchased from Anpel Laboratory Technologies (Shanghai, China). Fatty acid methyl ester (FAME) standards were sourced from multiple suppliers: Nu-Chek Prep (Elysian, MN, USA), Toronto Research Chemicals (TRC; Toronto, ON, Canada), ZZStandard (Shanghai, China), Larodan (Solna, Sweden), Aladdin (Shanghai, China), Sigma-Aldrich (St. Louis, MO, USA), and AccuStandard (New Haven, CT, USA).

### Lipid analysis

Milk samples were thawed at 4 °C and vortexed briefly for 10 s. For lipid extraction, a 20 μL aliquot of milk was mixed with 1 mL of dichloromethane–methanol (2:1, v/v) and vortexed for 15 min. Subsequently, 200 μL of water was added and the mixture was vortexed for an additional 1 min. Following centrifugation at 12,000 rpm for 10 min, 250 μL of the lower organic phase was collected and evaporated to dryness under a stream of nitrogen. The dried residue was reconstituted in 200 μL of acetonitrile–isopropanol (1:1, v/v) for LC–MS analysis. A pooled quality control (QC) sample was prepared by combining 10 μL from each individual milk sample.

Chromatographic separation was performed on a SCIEX ExionLC AD system coupled to an X500R QTOF mass spectrometer. A Waters ACQUITY CSH C18 column (1.7 μm, 2.1 × 100 mm) maintained at 45 °C was employed. Mobile phase A consisted of acetonitrile–water (60:40, v/v) with 0.1% formic acid and 10 mmol/L ammonium formate, while mobile phase B was acetonitrile–isopropanol (10:90, v/v) containing the same modifiers. The following gradient elution program (A/B, v/v) was applied: 0–1 min, 85:15; 3 min, 70:30; 3.5 min, 52:48; 12 min, 18:82; 12.5–17.5 min, 1:99; 17.6–20 min, 85:15. The flow rate was set to 0.4 mL/min and the injection volume was 2 μL.

Mass spectrometric detection was carried out in data‑dependent acquisition (IDA) mode. In positive ion mode, source parameters were as follows: ion source gas 1, 55 psi; gas 2, 55 psi; curtain gas, 35 psi; CAD gas, 7; source temperature, 450 °C; and spray voltage, 5500 V. MS scans were acquired over *m/z* 50–1200 with a declustering potential (DP) of 80 V, collision energy (CE) of 10 V, and an accumulation time of 100 ms. MS/MS scans were performed over the same mass range with a DP of 80 V, CE of 45 V, and an accumulation time of 50 ms. IDA criteria included a maximum of 10 candidate ions, an intensity threshold >500 cps, dynamic background subtraction, and exclusion of former candidate ions for 5 s after one occurrence. In negative ion mode, the source temperature was increased to 500 °C and the spray voltage was set to –4500 V; DP and CE values were –80 V and –10 V for MS, and –80 V and –45 V for MS/MS. The IDA intensity threshold was >1000 cps with an exclusion time of 6 s after one occurrence. All other parameters remained identical to those used in positive mode. Mass accuracy was ensured by automatic calibration using the SCIEX CDS (Calibration Delivery System) solution before each analytical sequence and after every five samples, yielding a root‑mean‑square (RMS) error of <±2ppm in both MS and MS/MS modes. A QC sample was injected every nine samples to monitor analytical repeatability.

Raw data files (.wiff) were converted to .abf format using AbfConverter (Reifycs). Data processing, including peak detection, alignment, identification, gap filling, and normalization, was conducted with MS‑DIAL (version 4.17). Key processing parameters were as follows. For data collection, MS1 and MS2 mass tolerances were set to 0.01 Da and 0.025 Da, respectively. Peak detection employed a minimum peak height of 1000 amplitude and a mass slice width of 0.1 Da. MS/MS deconvolution used a sigma window value of 0.5 and an abundance cut‑off of 10 amplitude. Lipid identification was performed against the built‑in MSP spectral library with a retention time tolerance of 100 min, MS1 tolerance of 0.01 Da, MS2 tolerance of 0.05 Da, and an identification score cut‑off of 60%. Adduct ions considered were [M + H]⁺, [M + NH₄]⁺ and [M + H − H₂O]⁺ for positive mode, and [M − H]⁻ and [M + HCOO]⁻ for negative mode. Alignment parameters included a retention time tolerance of 0.05 min and an MS1 tolerance of 0.015 Da. Gap filling was performed. The lipid abundance matrix was used for all subsequent statistical analyses.

### TG extraction

Milk samples were thawed at 4 °C and vortexed for 10 s. A 1 mL aliquot of milk was mixed with 6 mL of dichloromethane–methanol (2:1, v/v) and vortexed for 15 min. After adding 1.5 mL of water, the mixture was vortexed for an additional 1 min and then centrifuged at 12,000 rpm for 10 min. The lower organic phase was collected, and the remaining aqueous phase was re‑extracted with 6 mL of dichloromethane–methanol (2:1, v/v). The two organic extracts were combined and evaporated to dryness under a nitrogen stream. The dried milk fat residue was reconstituted in 200 μL of dichloromethane–methanol (2:1, v/v).

TG was isolated from the total lipid extract by sequential SPE according to Teng et al.^49^ with minor modifications. An NH₂ SPE cartridge was preconditioned with 6 mL of hexane. The reconstituted milk fat extract was loaded onto the cartridge, and the neutral lipid fraction was eluted with 6 mL of dichloromethane–isopropanol (2:1, v/v). The eluate was dried under nitrogen and redissolved in 200 μL of dichloromethane–methanol (2:1, v/v). A Si SPE cartridge was then preconditioned with 6 mL of hexane. The neutral lipid fraction was applied to the cartridge, and the TG fraction was eluted with 6 mL of diethyl ether–hexane (1:9, v/v). The purified TG fraction was dried under nitrogen and stored at −80 °C until further analysis.

### TG FA analysis

Fatty acids esterified to the TG backbone were analyzed according to Chen et al.^50,51^. The purified TG fraction (obtained as described in Section TG extraction) was dissolved in 1 mL of hexane, and 200 μL of a C19:0 TG internal standard solution (5 mg/mL) was added. The sample was sequentially derivatized: first with 2 mL of 2% (w/v) methanolic NaOH at 50 °C for 20 min, followed by 2 mL of 10% (v/v) acetyl chloride in methanol at 90 °C for 150 min. After cooling, 10 mL of saturated sodium chloride solution was added, and the upper organic phase containing the FAMEs was collected and diluted with hexane as required.

FAME analysis was performed on an Agilent 7890A gas chromatograph coupled to an Agilent 5975C mass spectrometer. Separation was achieved using an Agilent CP‑Sil 88 capillary column (100 m × 0.25 mm × 0.20 μm). Data were acquired in selected ion monitoring (SIM) mode following previously established parameters^50,51^.

Quantification and unit conversion. Raw GC–MS data were processed with MassHunter Quantitative software (version 10.0.707.0, Agilent). Chromatographic peaks were automatically integrated and then verified manually. FAMEs were identified by comparing retention times and ion abundance ratios with those of authentic standards. Quantification employed the internal standard method. Because SIM response depends on FA chain length and degree of unsaturation, individual calibration curves were constructed for each FAME using mixed standard solutions containing known concentrations of the analyte and the C19:0 FAME internal standard. Compound‑specific response factors derived from these curves were used to determine the mass concentration of each FAME. The corresponding FA mass concentrations were then calculated using established conversion factors. Using the molar mass of each FA, molar concentrations were obtained. Finally, the molar percentage (mol%) of each FA relative to total FAs was computed by dividing its molar concentration by the sum of all quantified FA molar concentrations.

### Sn-2 fatty acid analysis

Sn-2 MGs were generated from milk fat TGs by regiospecific hydrolysis according to Yener et al.^31^ with minor modifications. The purified TG fraction (approximately 100 mg), obtained as described in Section TG extraction, was dissolved in 2 mL of ethanol, and 44 mg of Candida antarctica lipase B (Novozym 435) was added. The mixture was incubated at 30 °C for 10 min and then shaken horizontally at 170 rpm and 30 °C for up to 3 h. The lipase was subsequently removed by filtration through absorbent cotton, and the hydrolysate was evaporated to dryness under a nitrogen stream. The dried residue was reconstituted in 200 μL of dichloromethane–methanol (2:1, v/v).

A Si SPE cartridge was preconditioned with 6 mL of hexane. After loading the hydrolysate, the cartridge was eluted sequentially with 6 mL of hexane, 6 mL of hexane–diethyl ether (95:5, v/v), 6 mL of hexane–diethyl ether (90:10, v/v), and finally 12 mL of methanol to collect the 2‑MG fraction. The 2‑MG fraction was dried under nitrogen and analyzed for fatty acid composition following the same derivatization and GC–MS procedure described in Section TG FA analysis.

FA composition at the sn‑2 and sn‑1(3) positions was determined according to the intra‑positional and inter‑positional distribution methods of Yener et al.^31^. Intra‑positional compositions are expressed as mol% of FAs within the respective position. The sn‑1(3) intra‑positional composition (mol%) was derived from the total TG FA profile and the sn‑2FA profile using Eq. (1):

1 $${\rm{Intra}}\;{\rm{sn}}-1(3){{\rm{FA}}}_{i}({\rm{mol}} \% )=\frac{3\times {\rm{TG}}{{\rm{FA}}}_{i}-{\rm{Intra}}\;{\rm{sn}}-2{{\rm{FA}}}_{i}}{2}$$

The inter‑positional distribution— the proportion of a given fatty acid that resides at a specific position relative to its total abundance across all three sn‑positions—was calculated using Eqs. (2) and (3).

2 $$\mathrm{Inter\; sn}-2{\mathrm{FA}}_{{\rm{i}}}=\frac{\mathrm{Intra\; sn}-2{\mathrm{FA}}_{{\rm{i}}}}{\mathrm{Intra\; sn}-2{\mathrm{FA}}_{i}+2\times \mathrm{Intra\; sn}-1(3){\mathrm{FA}}_{i}}$$

3 $$\mathrm{Inter\; sn}-1(3){\mathrm{FA}}_{i}=\frac{\mathrm{Intra\; sn}-1(3){\mathrm{FA}}_{i}}{\mathrm{Intra\; sn}-2{\mathrm{FA}}_{i}{\rm{i}}+2\times \mathrm{Intra\; sn}-1(3){\mathrm{FA}}_{i}\,}$$

### Lipid similarity evaluation

A similarity evaluation model was established using the Bray–Curtis similarity index^52^. The similarity evaluation indicators (SEIs) were divided into three condition groups: (i) total FA composition, (ii) sn‑2 FA composition, and (iii) lipid molecular species. The overall similarity scores of eight mammalian milks relative to human milk were calculated using the following formulas (4)–(8):

4 $${\rm{G}}=\frac{{{\rm{G}}}_{\mathrm{FA}}+{{\rm{G}}}_{\mathrm{sn}-2\mathrm{FA}}+\,{{\rm{G}}}_{\mathrm{lipid\; molecularspecies}}}{3}\,$$

5 $${{\rm{G}}}_{\mathrm{FA}}=\frac{\mathrm{SEI}\left(\,{\mathrm{FA}}_{1}\right){+}_{\ldots \ldots .}+\mathrm{SEI}({\mathrm{FA}}_{{\rm{n}}})}{{\rm{n}}}\,$$

6 $${{\rm{G}}}_{sn-2\mathrm{FA}}=\frac{\mathrm{SEI}(sn-2{\mathrm{FA}}_{1}){+}_{\ldots \ldots .}+\mathrm{SEI}(\mathrm{sn}-2{\mathrm{FA}}_{{\rm{n}}})}{{\rm{n}}}$$

7 $${{\rm{G}}}_{\mathrm{lipid\; molecular\; species}}=\frac{\mathrm{SEI}({\mathrm{lipid\; molecular\; species}}_{1}){+}_{\ldots \ldots .}+\mathrm{SEI}({\mathrm{lipid\; molecular\; species}}_{{\rm{n}}})}{{\rm{n}}}\,$$

8 $$\mathrm{SEI}(\mathrm{lipid})=\frac{2\times f}{f+F}$$

Where *G* is the total similarity score of the tested mammalian milk relative to human milk, with a maximum value of 1. *G*_FA_、*G*_sn-2 FA_、*G*_lipid molecular species_ are the scores for total FA composition, sn‑2 FA composition, and lipid molecular species, respectively. *f* is the minimum value of a given component between human milk and the mammalian milk. *F* is the maximum value of that component between human milk and the mammalian milk.

### Statistical analysis

Data were preliminarily organized using Microsoft Excel. All statistical analyses and visualizations were conducted in R (version 4.4.1), Python (version 3.6.6), and Origin 2021. Normality of FA and lipid molecular abundance data was assessed using the Shapiro–Wilk test, and homogeneity of variances was evaluated with Bartlett’s test. Detailed results of these assumption checks for each dataset are provided in Supplementary Data 3 and 4.

For pairwise comparisons of lipid molecular species between each mammalian milk and human milk, differential lipids were screened based on VIP > 1, *P* < 0.05, and FC > 2 or <0.5. When data met the assumptions of normality and homoscedasticity, Student’s t‑test was applied; otherwise, the Wilcoxon rank‑sum test was used. These analyses were performed using the MetaboAnalystR package in R.

For multi‑group comparisons of total TG FA composition, sn‑2 positional FAs, and sn‑1(3) positional FAs, one‑way analysis of variance (ANOVA) followed by Tukey’s honestly significant difference (HSD) post‑hoc test was employed when data were normally distributed and variances were homogeneous. When these assumptions were violated, the Kruskal–Wallis test followed by Dunn’s post‑hoc test with Benjamini–Hochberg correction was applied. These analyses were also implemented using MetaboAnalystR. Positional preference of a fatty acid for the sn‑2 or sn‑1(3) position was tested against the theoretical random value of 33.3% using a one‑sample t‑test. Significance was set at *P* < 0.05 for all statistical tests.

PCA was performed using the prcomp function from the stats package in R. Bar plots and donut charts were generated with ggplot2. Venn diagrams were drawn using VennDiagram. Clustered heatmaps were constructed with circlize. Sankey diagrams and volcano plots were created in Python using the pandas library. Radar plots were produced in Origin 2021.

## Supplementary information

Supplementary data

Supplementary data

## Data Availability

The datasets generated during the current study are available from the corresponding author on reasonable request.
