# Supplementary material for: Integrated lipidomics and fatty acid positional distribution for nutritional assessment of nine mammalian milks
Source: NPJ Sci Food. 2026 Jul 3;10:329. doi: 10.1038/s41538-026-00954-w (PMC13696699; doi:10.1038/s41538-026-00954-w)
Supplement: Supplementary file 1 [file 41538_2026_954_MOESM1_ESM.pdf]

Supplementary Figure 1 OPLS-DA score plots in pairwise comparisons between eight mammalian milks and human milk. (A) Cow *vs* Human Milk (B) Buffalo *vs* Human Milk (C) Yak *vs* Human Milk (D) Goat *vs* Human Milk (E) Sheep *vs* Human Milk (F) Camel *vs* Human Milk (G) Donkey *vs* Human Milk (H) Mare *vs* Human Milk.

Supplementary Data 1 Lipid molecular species identified in milk from nine mammalian species

Supplementary Data 2 Differential lipids in pairwise comparisons between human milk and milk from different mammalian species

Supplementary Data 3 Normality and homogeneity of variance for lipid molecules

Supplementary Data 4 Normality and homogeneity of variance for fatty acids

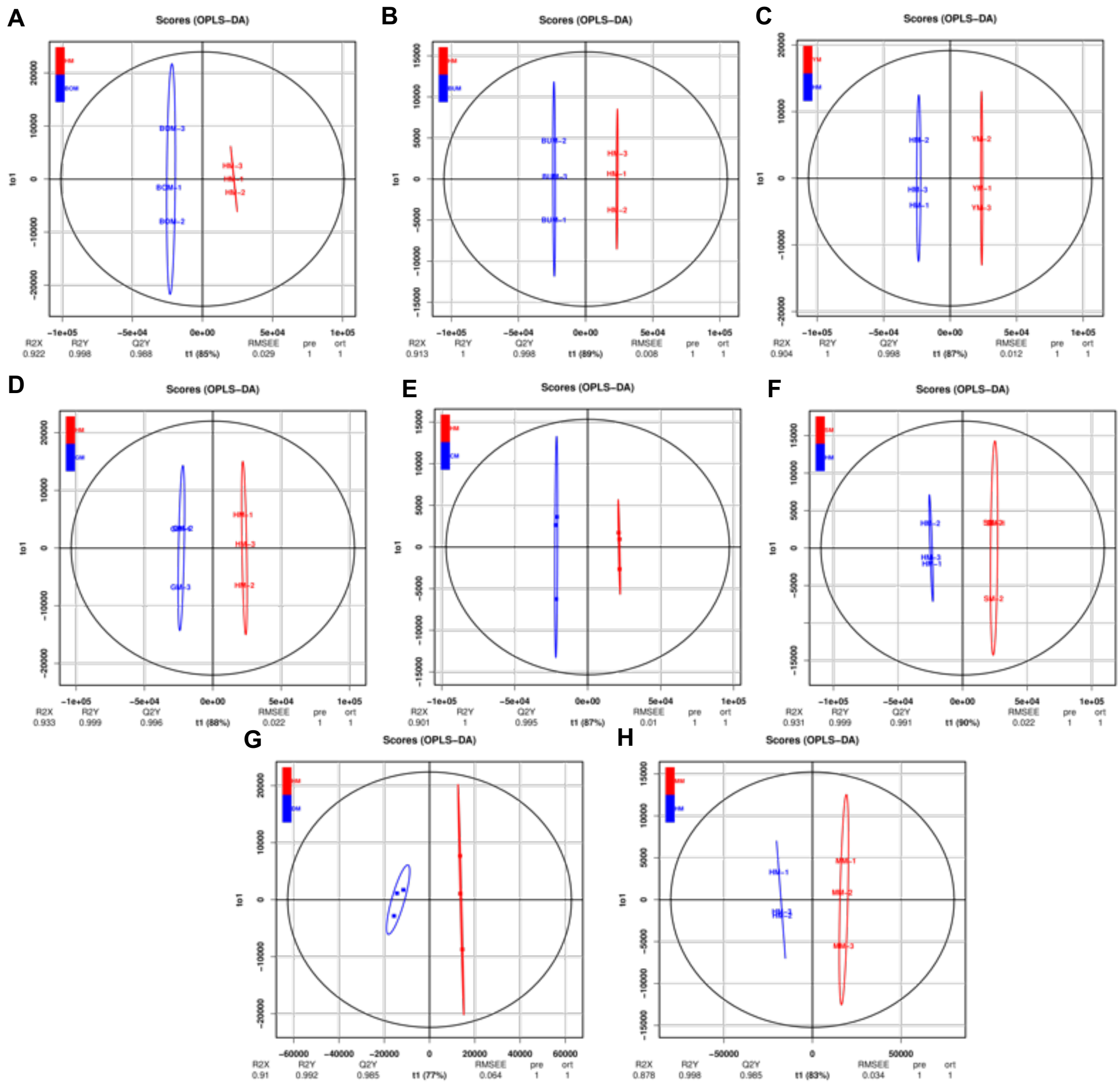

Supplementary Figure 1 OPLS-DA score plots in pairwise comparisons between eight mammalian milks and human milk. (A) Cow *vs* Human Milk (B) Buffalo *vs* Human Milk (C) Yak *vs* Human Milk (D) Goat *vs* Human Milk (E) Sheep *vs* Human Milk (F) Camel *vs* Human Milk (G) Donkey *vs* Human Milk (H) Mare *vs* Human Milk.
